# Unveiling the Miniband Structure of Graphene Moiré Superlattices via Gate-Dependent Terahertz Photocurrent Spectroscopy

**DOI:** 10.1021/acsnano.5c05306

**Published:** 2025-07-21

**Authors:** Juan A. Delgado-Notario, Stephen R. Power, Wojciech Knap, Manuel Pino, JinLuo Cheng, Daniel Vaquero, Takashi Taniguchi, Kenji Watanabe, Jesús E. Velázquez-Pérez, Yahya Moubarak Meziani, Pablo Alonso-González, José M. Caridad

**Affiliations:** † Departamento de Física Aplicada, 16779 Universidad de Salamanca, 37008 Salamanca, Spain; ‡ School of Physical Sciences, 8818 Dublin City University, Glasnevin, Dublin 9, Ireland; § CENTERA Laboratories, Institute of High Pressure Physics, Polish Academy of Sciences, Warsaw 01-142, Poland; ∥ Centre for Advanced Materials and Technologies CEZAMAT, Warsaw University of Technology, Warsaw 02-822, Poland; ⊥ Departamento de Física Fundamental (IUFFyM) y GIR Nanotecnología, Universidad de Salamanca, 37008 Salamanca, Spain; # GPL Photonics Laboratory, State Key Laboratory of Luminescence and Applications, Changchun Institute of Optics, Fine Mechanics and Physics, Chinese Academy of Sciences, Changchun, Jilin 130033, People’s Republic of China; ∇ University of Chinese Academy of Sciences, Beijing 100039, China; ○ Zernike Institute for Advanced Materials, University of Groningen, 9747 AG Groningen, The Netherlands; ◆ Research Center for Electronic and Optical Materials, 52747 National Institute for Materials Science, Tsukuba 305-0044, Japan; ¶ Department of Physics, University of Oviedo, Oviedo 33006, Spain; & Unidad de Excelencia en Luz y Materia Estructurada (LUMES), Universidad de Salamanca, Salamanca 37008, Spain

**Keywords:** terahertz, graphene, two dimensional materials, moiré superlattices, spectroscopy, miniband structure

## Abstract

Moiré superlattices formed at the interface between stacked 2D atomic crystals offer limitless opportunities to design materials with widely tunable properties and engineer intriguing quantum phases of matter. However, despite progress, precise probing of the electronic states and tantalizingly complex band textures of these systems remain challenging. Here, we present gate-dependent terahertz photocurrent spectroscopy as a robust technique to detect, explore, and quantify intricate electronic properties in graphene moiré superlattices. Specifically, using terahertz light at different frequencies, we demonstrate distinct photocurrent regimes, evidencing the presence of avoided band crossings and tiny (∼1 to 20 meV) inversion-breaking global and local energy gaps in the miniband structure of minimally twisted graphene and hexagonal boron nitride heterostructures, key information that is inaccessible by conventional electrical or optical techniques. In the off-resonance regime, when the radiation energy is smaller than the gap values, enhanced zero-bias responsivities arise in the system due to the lower Fermi velocities and specific valley degeneracies of the charge carriers subjected to moiré superlattice potentials. In stark contrast, the above-gap excitations give rise to bulk photocurrents—intriguing optoelectronic responses related to the geometric Berry phase of the constituting electronic minibands. Besides their fundamental importance, these results place moiré superlattices as promising material platforms for advanced, sensitive, and low-noise terahertz detection applications.

## Graphical abstract

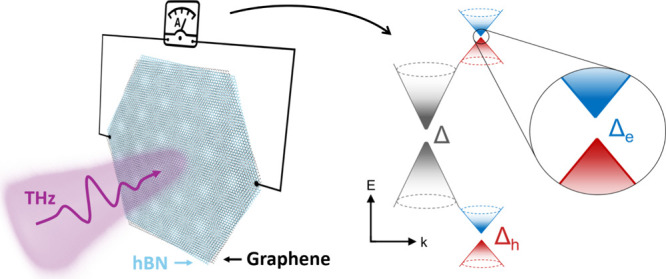

## Introduction

In van der Waals (vdW) heterostructures, lattice mismatch and rotation between adjacent crystals can lead to the formation of a moiré superlattice with a periodicity larger than the atomic scale. Such periodic potentials induce notable changes in the electronic, optical, and mechanical properties of different two-dimensional (2D) crystals,^1–3^ and may prompt the appearance of a number of exotic fundamental phenomena. In the case of graphene-based moiré superlattices, some of these effects include, for instance, superconductivity,^4^ magnetism^5^ or correlated insulator phases.^6^ In addition, moiré structures have recently been shown to be an interesting material platform for the realization of different electronic components such as single-electron transistors,^7^ superconducting quantum interference devices^8^ or efficient visible/infrared photodetectors.^9,10^

According to theoretical predictions,^11–13^ graphene moiré superlattices are also expected to be unique systems to design state-of-the-art, compact optoelectronic devices working at terahertz (THz) frequencies.^14,15^ Moreover, measurements in this frequency range also have the potential to act as a diagnostic tool, providing valuable and elusive information about the unique and convoluted electronic structure of moiré superlattice systems^13,16–18^ which ultimately depends on the subtle atomic registry and interactions existing between the layered materials.

A relevant example is the absence or presence (and possible quantification) of avoided band crossings and tiny (<10 meV) energy gaps in the conduction band states of elementary moiré superlattices made of graphene and hexagonal boron nitride, intriguing features which have remained under debate up to now.^2,19,20^ In this sense, a key open question is whether such energy gaps do exist but have not been experimentally observed due to the insufficient sensitivity and resolution of the applied experimental methods and diagnostic tools so far.

In the present work, we combine THz photocurrent spectroscopy (excitation frequencies between 0.075 and 4.7 THz) with the continuous tuning of the Fermi level via gate voltage to explore and probe the band structure singularities and intricate electronic states of graphene moiré superlattices. Our samples were fabricated by aligning the crystal lattices of graphene and hexagonal boron nitride (hBN) at rotation angles θ < 2° (Figure 1a). Interestingly, we show that the gate-tunable THz photocurrent collected in these devices can originate from intraband or interband transitions, depending on the excitation frequency, and such responses are exquisitely sensitive to very fine details of the electronic structure of graphene in the presence of moiré potentials. Specifically, THz photocurrent measurements at multiple frequencies provide important information about the miniband structure appearing in the vicinity of the $\overset{-}{K}$ / $\overset{-}{K^{\prime }}$ and Γ points of the superlattice Brillouin zone, featuring a new generation of Dirac Fermions, so-called secondary or superlattice Dirac points, sDPs^1–3^ (Figure 1b), as well as other important aspects such as the reduced Fermi velocity of charge carriers near sDPs with respect to the main Dirac point, DP,^21^ or the presence and size of energy gaps at the valence band satellite Dirac point^2^ (Δ_h_). More intriguingly, we also demonstrate how gate-tunable THz photoresponse measurements of graphene moiré superlattices at multiple frequencies are able to detect the presence of tiny local energy gaps (sizes of a few meV) at the conduction band satellite Dirac point (Δ_e_). These are subtle features “hidden” to conventional probing techniques such as quantum transport measurements^2,20^ or angle-resolved photoemission spectroscopy (ARPES),^22^ experimental methods which can only probe overall gaps (i.e., bandgaps) existing in these quantum materials or are limited to an energy resolution of ∼30 meV, respectively. In addition, we unveil that the recorded photoresponses are, furthermore, sensitive to quantum geometric textures of the gapped states occurring at the miniband satellite Dirac points.

**Figure 1:**
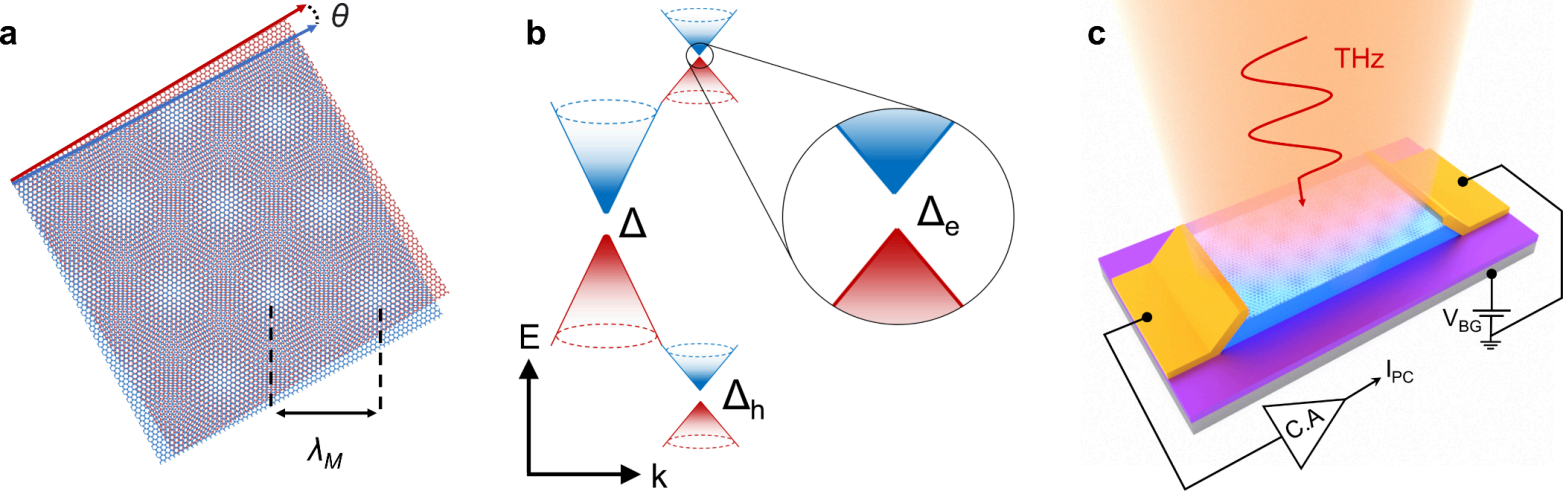
Moiré THz devices. (a) Illustration of a moiré pattern with long wavelength λ_M_, generated by aligning graphene (red) and hBN (blue) honeycomb lattices by an angle θ below 2 degrees. (b) Schematic of the band structure of the graphene/hBN moiré superlattices featuring a new generation of Dirac Fermions (so-called secondary or superlattice Dirac points, sDPs), the appearance of gaps at the main (Δ) and superlattice Dirac points (Δ_e_ and Δ_h_) and the different Fermi velocity of charge carriers close to each of the Dirac points (i.e., different slope of the Dirac cones).^21^ (c) Schematic of the zero drain-bias photocurrent (*I*_PC_) measurements taken at different THz frequencies.

As such, the gate- and frequency-dependent THz photoresponse of graphene moiré materials is demonstrated to be a complete and sensitive technique to accurately probe and disclose relevant and exclusive information about the intricate electronic structure of graphene’s Dirac electrons subjected to superlattice potentials. This outstanding ability is of particular significance given the fact that current fabrication methods suffer from distortions (due to random strain or the control of the twist angle^23^) that impact the electronic properties of graphene moiré superlattices. From a more technological point of view, samples where the crystal lattices of graphene and hBN are close to perfect alignment (θ = 0°) show enhanced zero drain-bias responsivity values close to the sDPs with respect to the main DP, and thus this work also paves the way toward engineering moiré superlattice-based THz detectors with high-speed, low-noise, and extreme sensitivity.

## Results and Discussion

### Fabrication of Moiré THz Devices

Following published techniques for the exfoliation and vdW assembly of 2D materials,^24,25^ we assemble (Methods and Supporting Information Note 1) several graphene heterostructures, where both the graphene (monolayer, MLG, or bilayer, BLG) and hBN lattices are aligned by a rotation angle θ < 2° (see Figure 1a). We anticipate that the use of monolayer or bilayer graphene crystals is equivalent for this study (see electrical and photocurrent measurements described below).

We then fabricate five different devices and measure their zero drain-bias photocurrent at THz frequencies (see Figure 1c and Methods). The top-left inset of Figure 2a shows one of these devices (here named device A), consisting of a conventional short-channel (SC), dual-gated architecture.^26–28^ Besides, we measure four additional devices shaped in a similar SC architecture (device B) or other different and well-known geometries (e.g., multicross bars^14^ (MC), device C, or interdigitated dual-gated transistors^29^ (IDGT), devices D and E, see details in Supporting Information Note 2) to ensure that our conclusions are generic to the effect of moiré potentials in graphene and not dependent on a specific device architecture.

**Figure 2:**
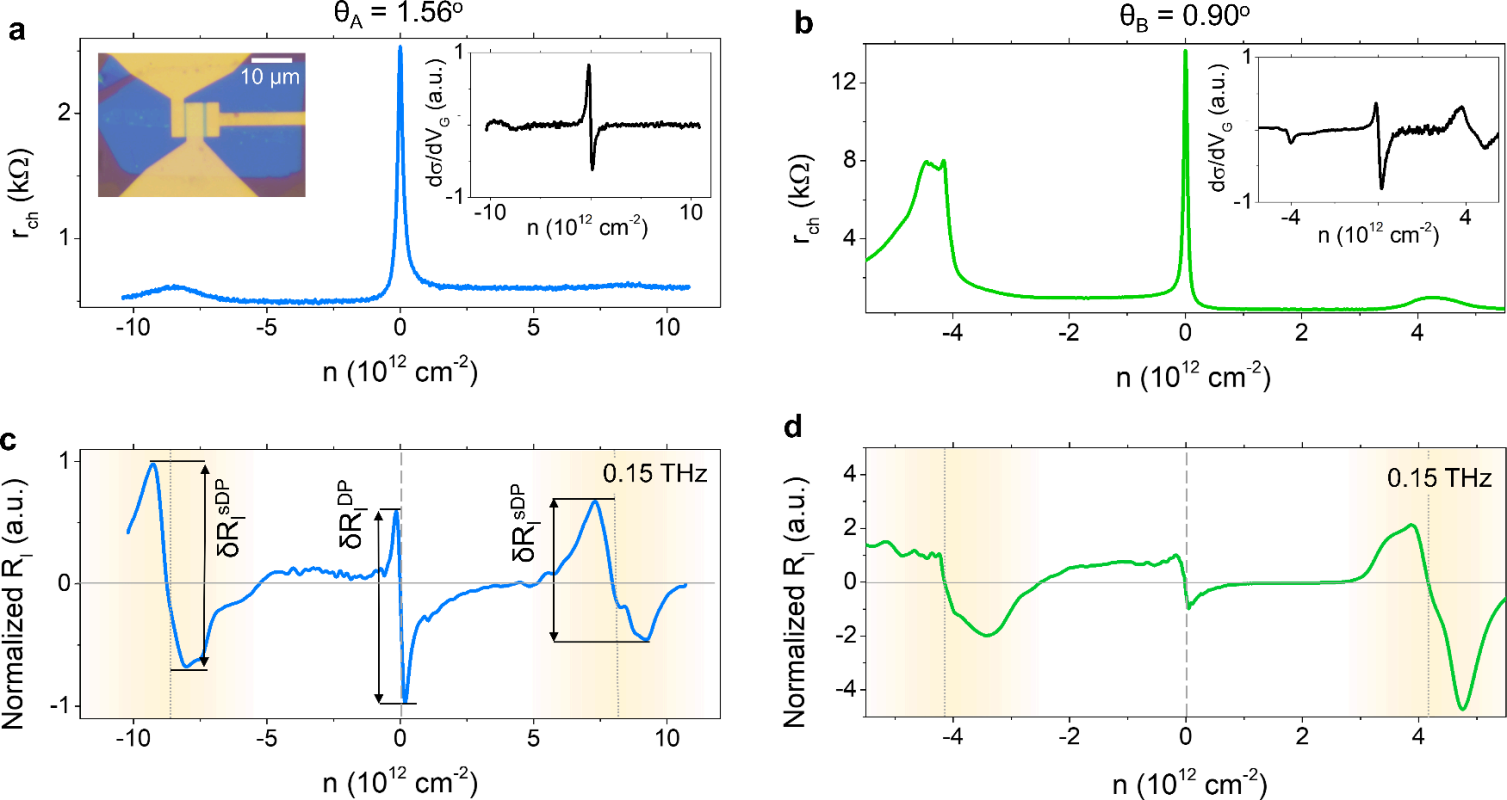
Electrical and optoelectronic characteristics of graphene moiré devices. (a) Measured channel resistance, *r*_ch_, as a function of the carrier density, *n*, for device A at 10 K. Top left inset shows an optical photograph of a SC device. (b) Measured channel resistance, *r*_ch_, as a function of the carrier density, *n*, for device B at 10 K. Top right insets in panels (a) and (b) show the variation of the channel conductivity σ with respect to the carrier density, dσ/d*n*, as a function of *n*. (c) Measured photocurrent responsivity *R*_*I*_ as a function of the carrier density, *n*, for device A at 10 K under the excitation of 0.15 THz. (d) Measured photocurrent responsivity *R*_*I*_ as a function of the carrier density, *n*, for device B at 10 K under the excitation of 0.15 THz. Vertical lines mark the positions of the main (dashed) and satellite (continuous) Dirac points.

### Electrical Characterization of the Devices

We first measured and assessed the transport characteristics of the fabricated devices (measurement details shown in Methods). All these systems are of high quality, with carrier mobilities well exceeding 150,000 cm^2^ V^–1^ s^–1^ at low temperatures *T* (see Supporting Information Note 3). Figure 2 depicts the measured channel resistance, r_ch_, as a function of the carrier density, *n*, for the SC devices of the study, devices A (Figure 2a) and B (Figure 2b) at 10 K. The corresponding data for devices C–E are shown in Supporting Information Note 2. In agreement with previous transport measurements of MLG and BLG coupled to a moiré superlattice,^2,3^ *r*_ch_ in all these devices exhibits three resistance peaks at carrier densities 0 cm^–2^ and ±|*n*_SP_| (the latter positioned around ∼±8 × 10^12^ and ∼±4 × 10^12^ cm^–2^ for devices A and B, respectively). Such features are the result of a depression in the density of states occurring in the electronic band structure of these systems at the DP and the two sDPs (in both conduction and valence bands), respectively.

By using the charge density at which these satellite resistance peaks are observed *n*_SP_, one can estimate both the moiré wavelength, λ_M_, and the relative rotation angle, θ, between the graphene and hBN lattices in the devices.^1–3^ In particular, λ_M_^(A)^ = 7.6 nm, θ_A_ = 1.56°, and λ_M_^(B)^ = 10.4 nm, θ_B_ = 0.9° for devices A and B, respectively. The details of these calculations as well as the values of λ_M_ and θ extracted for all devices A–E are shown in Supporting Information Note 4. In general, θ lies between 0.4 and 1.6° in all studied devices. We note that many of our devices show long wavelength values which are not much smaller than the expected maximum around 14 nm,^1,2^ which implies a nearly perfect rotational alignment of the graphene and hBN crystals with θ < 1° and may result in a commensurate state of graphene on hBN (local enlargement of the lattice constant of graphene to match the one of hBN).

### THz Photodetection in Graphene Moiré Superlattice Devices

Figure 2c,d shows the current responsivity, *R*_*I*_ = *I*_PC_/*P*, of the fabricated moiré THz detectors A and B, respectively, as a function of *n* measured at 0.15 THz and a temperature of 10 K (*R*_*I*_ of devices C–E can be found in Supporting Information Note 5). Here, *I*_PC_ is the collected zero drain-bias photocurrent (Figure 1c), and *P* is the incident radiation power. For clarity, *R*_*I*_ is normalized with respect to the maximum photocurrent value measured close to the main Dirac point. At a low chemical potential *E*_F_, in the vicinity of the main DP (doping level interval |*n*| < 2 × 10^12^ cm^–2^), the photoresponse in the studied devices exhibits a different sign which depends on the type of charge carrier existing in the device channel, electrons or holes. The sign reversal of the photocurrent occurs right at the DP, and the photoresponse tends to zero at both ends of the considered interval, i.e., at values *n* ∼ ±2 × 10^12^ cm^–2^. This qualitative behavior is consistent with the one reported for THz photodetectors made of bare graphene,^28–31^ and stems from the ambipolar charge transport present in this material, indicating the dominant role of intraband absorption in the photocurrent *I*_PC_.^28–30^ Interband transitions would result in the generation of electron–hole pairs, and the variation of the responsivity with respect to *n* should have yielded a photocurrent peak at the Dirac point,^14^ instead. In particular, intraband-type photoresponse has a functional dependence on *n* which is proportional to dσ/d*n* (see inset panels of Figure 2a,b), with σ being the dc conductivity of the material. There are two physical intraband phenomena that may give rise to such type of photoresponse in graphene devices at THz frequencies: plasma wave rectification and photothermoelectric effects.^30^ The fact that the photoresponse *R*_*I*_ tends to vanish at large *n* (i.e., for Fermi levels *E*_F_ sufficiently separated from the main Dirac point) indicates that plasma wave rectification is the dominant mechanism in our devices, rather than photothermoelectric effects.^26–28^

In the following, we examine the measured photoresponse at higher *E*_F_, close to the superlattice Dirac points (carrier densities ±|*n*_SP_|, see highlighted region in Figure 2c,d). In particular, we demonstrate that the line shape and magnitude of the photocurrent responsivity *R*_*I*_(*n*) at different frequencies are sensitive to many of the key characteristics of the miniband structure of graphene moiré superlattice systems,^32^ including (i) the presence of superlattice Dirac points, (ii) the reduced Fermi velocity and distinct valley degeneracy of charge carriers near sDPs with respect to the main Dirac point, and even (iii) the complex band texture occurring at *E*_F_ close to the sDPs, including several avoiding band crossings giving rise to a series of global and local energy gaps.

#### Presence of Superlattice Dirac Points

The overall measured photocurrent response around the position of the satellite Dirac points ±|*n*_SP_| at 0.15 THz exhibits a qualitative trend that is similar to the one reported close to the main Dirac point. In particular, *R*_*I*_(*n*) changes sign right at the sDPs both in the hole and in the electron bands, −|*n*_SP_| and +|*n*_SP_|, respectively. Such behavior has been already observed^26^ and is consistent with the existence of electron–hole pockets located near the $\overset{-}{K}$ / $\overset{-}{K^{\prime }}$ and Γ-points of the superlattice Brillouin zone^32^ (i.e., superlattice Dirac points) in both the valence and conduction bands (Figure 1b) and the generation of plasmons in such superlattice minibands.^12^

#### Reduced Fermi Velocity and Valley Degeneracy of Charge Carriers near sDPs

We then examine the measured photoresponses at a more quantitative level. Figure 2c shows that, in our device with θ ∼ 1.56° (device A), the *R*_*I*_ signal near the two sDPs is similar in magnitude to that measured near the main DP. In contrast, devices with θ < 1° (devices B–E, see Figure 2d and Supporting Information Note 5) present an enhanced *R*_*I*_ around the two sDPs compared to the signal measured near the main DP. We quantify such enhancement by taking into account the photocurrent responsivity variation close to both main (δ*R*_*I*_^DP^) and satellite Dirac points (δ*R*_*I*_^sDP^), where δ*R*_*I*_^DP^ and δ*R*_*I*_^sDP^ are the differences in the measured maxima and minima of *R*_*I*_ around carrier densities 0 cm^–2^ and ±|*n*_SP_|, respectively (see marks in Figure 2c). Overall, enhancement ratios δ*R*_*I*_^sDP^/δ*R*_*I*_^DP^ between 1.5 and 5 are observed in devices with θ < 1° at *T* = 10 K (Figure 2d and Supporting Information Note 5).

Some of these quantitative results are surprising at first glance. In particular, whereas the responsivity ratio δ*R*_*I*_^sDP^/δ*R*_*I*_^DP^ ≤ 1 observed in Figure 2c (device A, θ ∼ 1.56°) can be easily explained by the variation in the channel conductivity with respect to *n* (dσ/d*n*, see inset of Figure 2a); ratios δ*R*_*I*_^sDP^/δ*R*_*I*_^DP^ > 1 observed in Figure 2d and Supporting Information Note 5 (devices B–E, all with θ < 1°) cannot be understood from dσ/d*n* (see inset of Figure 2b and Supporting Information Note 6). The highlighted quantitative difference can be explained by the extraordinary sensitivity of the intraband photocurrent to the electronic Fermi surface of the system under study. In particular, we argue that the δ*R*_*I*_^sDP^/δ*R*_*I*_^DP^ > 1 measured in devices B–E can be explained by the larger density of states (DOS) present at energies around the sDPs in graphene/hBN heterostructures with small misalignment angles^1^ θ < 1°. Following a straightforward analysis (see Supporting Information Note 6), one can roughly estimate the responsivity enhancement in the zero-temperature and zero-carrier density limit to be

$$\frac{\delta R_{I}^{\mathrm{sDP}}}{\delta R_{I}^{\mathrm{DP}}}\approx \frac{(g_{v}^{\left(\mathrm{sDP}\right)}{)}^{3/2}v_{F}^{\left(\mathrm{DP}\right)}}{(g_{v}^{\left(\mathrm{DP}\right)}{)}^{3/2}v_{F}^{\left(\mathrm{sDP}\right)}}$$ 1

where *g*_*v*_ and *v*_F_ are the valley degeneracy and the Fermi velocity of charge carriers. These two band structure parameters are different near the main and satellite Dirac points,^1,32,33^ and such differences can explain the photocurrent enhancement observed near the satellite Dirac points in samples with θ < 1°. Indeed, whereas the valley degeneracy near the DP (*g*_*v*_^(DP)^) is 2, the value near the sDP (*g*_*v*_^(sDP)^) is 2 or 6 in common bandstructure reconstructions of graphene moiré superlattices. Moreover, the Fermi velocity of charge carriers at the satellite Dirac points *v*_F_^(sDP)^ in well aligned devices (θ < 1°) has been shown to be ∼0.50 to 0.73 times smaller than the Fermi velocity at the Dirac point *v*_F_^(DP)^. From the aforementioned values, one can estimate enhanced responsivity ratios δ*R*_*I*_^sDP^/δ*R*_*I*_^DP^ in graphene moiré superlattice devices at vanishing temperature and carrier density to approximately stand between 1 and 10. These calculations are in good agreement with our experiments, which report enhanced responsivity values between 1.5 and 5 in all our devices with θ < 1°, especially considering that our measurements are undertaken at finite temperatures (10 K) and that the residual doping in our samples is typically ∼10^11^ cm^–2^.

#### Existence and Size of Energy Gaps at the Satellite Dirac Points

The presence of moiré potentials breaks the inversion symmetry of the heterostructure, which, in principle, could lead to a gap opening at both the main and the two satellite Dirac points.^19,34,35^ Indeed, multiple electrical and optical measurements in the literature have provided convincing evidence of energy gaps present in aligned graphene/hBN devices at the main Dirac point (Δ) and the valence band satellite Dirac point (Δ_h_).^2,20,22,36^ The size of these gaps is ∼10 to 40 meV, with larger values occurring closer to perfect alignment (θ = 0°).^3^ However, despite predictions,^32,34,35,37,38^ no energy gaps have been observed up to now in the case of the conduction band sDP (Δ_e_).

We demonstrate that gate-dependent and zero-bias THz photodetection at different frequencies is a relevant technique to examine the presence of tiny ∼ meV global and local energy gaps at both satellite Dirac points Δ_h_ and Δ_e_ in graphene moiré superlattice systems. This is due to several reasons, including the fact that THz radiation covers an energy range, which is comparable to the size of these gaps. Moreover, zero-bias, gate-dependent THz photocurrents have different (intra- or interband) origins and thus exhibit well-differentiated features depending on the chemical potential *E*_F_ and the frequency of the incoming radiation.

For instance, at *E*_F_ close to the valence band sDP (Figure 3a), device A shows a clear transition from an intraband to an interband type of photodetection when increasing the radiation frequency from 0.075 to 4.7 THz (see Figure 3b). Specifically, whereas the measured photocurrent responsivity *R*_*I*_(*n*) is consistent with an intraband-type mechanism (exhibiting a vanishing photocurrent and a sign change right at the hole sDP) for excitation frequencies equal to or below 2.5 THz (≈10 meV), *R*_*I*_(*n*) changes at higher frequencies and exhibits a pronounced and stable minimum at the position of the sDP for excitation frequencies larger than 4.1 THz (≈17 meV), instead. The latter line shape is a clear indication that interband transitions become dominant at frequencies ≥4.1 THz and provides an estimate of the energy gap size, Δ_h_ ≈ 17 meV. Such value agrees well with the one extracted via traditional methods such as temperature-dependent transport measurements, both in literature^2^ and in our samples (see Supporting Information Note 7). On the other hand, the recorded zero-bias, gate-dependent THz photocurrents for Device B (Figure 3c) display an intraband-type behavior over the measurement range (0.075–4.7 THz), which is attributed to the fact that Δ_h_ in more aligned devices is larger^2^ than the highest radiation energy available in our experimental setup (∼20 meV)

**Figure 3:**
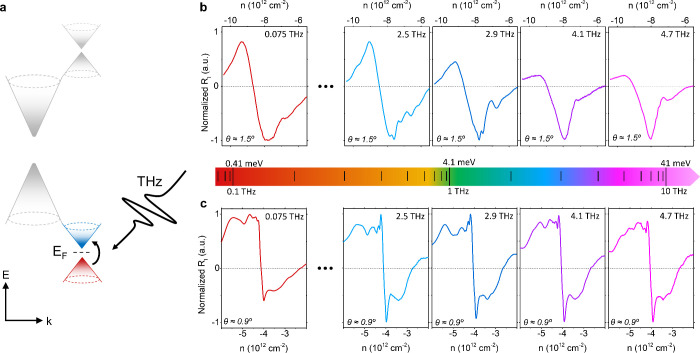
Low-temperature photocurrent spectroscopy measurements at the valence band sDP. (a) Schematic band structure of a graphene/hBN heterostructure, highlighting the possible presence of interband transitions at the hole-band sDP at THz frequencies. (b) Measured photocurrent responsivity *R*_*I*_ at different THz frequencies as a function of the carrier density, *n*, when the *E*_F_ is close to the valence-band sDP for Device A. (c) Measured photocurrent responsivity *R*_*I*_ at different THz frequencies as a function of the carrier density, *n*, when the *E*_F_ is close to the valence-band sDP for Device B. Measurements in panels (b) and (c) are undertaken at 10 K.

Importantly, gate-dependent THz photoresponses at multiple frequencies are also able to detect and assess the size of local energy gaps existing at the conduction band sDP (Δ_e_) in graphene moiré superlattices (Figure 4a). We remark that the existence of a nonzero gap Δ_e_ in graphene/hBN superlattices is an intriguing result which, despite predictions,^32,34,35,37,38^ has remained under debate up to now.^2,19,20^ In fact, conventional (optical and electrical) probing techniques^2,20,22^ have so far been unable to detect any gap at the conduction band sDP , and this is the main reason why several studies assume the absence of such energy gaps^2,20^ (further analysis is provided in the Discussion section). Figure 4b shows *R*_*I*_(*n*) measured close to the conduction-band sDP at different THz frequencies (from left to right *f* = 0.075, 0.15, 0.3, 2.5, and 4.7 THz, respectively) in device A (θ ∼ 1.6°). An interband type of photocurrent is present for frequencies ≥2.5 THz, and therefore, the energy gap Δ_e_ in this device is estimated to be ≈10 meV.

**Figure 4:**
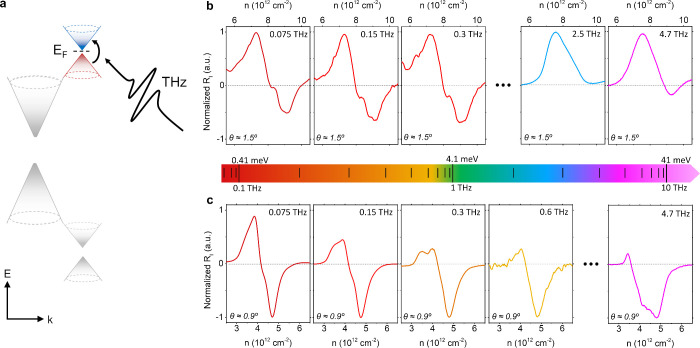
Low temperature photocurrent spectroscopy measurements at the conduction band sDP. (a) Schematic band structure of a graphene/hBN heterostructure, highlighting the possible presence of interband transitions at the electron-band sDP at THz frequencies. (b) Measured photocurrent responsivity *R*_*I*_ at different THz frequencies as a function of the carrier density, *n*, when the *E*_F_ is close to the conduction-band sDP for device A. (c) Measured photocurrent responsivity *R*_*I*_ at different THz frequencies as a function of the carrier density, *n*, when *E*_F_ is close to the conduction-band sDP for device B. Measurements in panels (b) and (c) are performed at 10 K.

In contrast, Figure 4c shows *R*_*I*_(*n*) measured close to the conduction-band sDP at different THz frequencies (from left to right *f* = 0.075, 0.15, 0.3, 0.6, and 4.7 THz, respectively) in device B. Whereas a clear photoresponse of intraband type is measured at *f* = 0.075 THz, a photoresponse of interband origin is already evident and stable at frequencies above 0.3 THz. This observation demonstrates the presence of a nonzero bandgap at the electron-band SDP in this device with θ ∼ 0.9° and suggests an approximate size of Δ_e_ ≈ 1.2 meV. By measuring and taking into account *R*_*I*_(*n*) close to the conduction band sDP in all devices under study (see Supporting Information Note 8), we corroborate that the size of Δ_e_ depends on the misalignment angle θ (see theoretical analysis below).

#### Theoretical Analysis

The results and interpretation given above are fully supported by calculations of the band structure and first- and second-order conductivities of these systems with a tight-binding model (Methods). Figure 5a,b depicts the simulated band structure of a representative graphene/hBN heterostructure near the valence and conduction band sDPs, respectively. The misalignment angle between graphene and hBN flakes in this simulation is set to θ = 0.44°, and thus the sDPs induced by the superlattice potential occur around energies^1^ *E*_sDP_ = ±π*v*_F_^(DP)^/λ_M_ ≈ ±150 meV (or equivalently, at carrier densities *n*_SP_ ≈ ±2.72 × 10^12^ cm^–2^), at the *K*, *K*’, and/or Γ points of the Brillouin zone of the rhombohedral supercell used for the calculation (see details in Supporting Information Note 9). Importantly, the band structure demonstrates that sDPs in both conduction and valence bands contain energy gaps, which are the result of Bragg scattering at the edges of the superlattice Brillouin zone.^39^ In particular, the band splitting is rather large on the hole side, leading to an actual spectral gap Δ_h_^1^ > 20 meV. We also highlight the fact that, similarly to other studies reported in literature,^35,38^ Figure 5b does not display a global band gap at the conduction band sDPs. Instead, we observe a complicated band texture, including a series of avoided band crossings, giving rise to several local energy gaps (marked Δ_e_^1^ to Δ_e_^4^) between consecutive bands. The smallest of these is ∼3 meV, an order of magnitude smaller than the hole side gap and in good agreement with measurements shown in Figure 4 for samples with θ < 1°. For clarity, Figure 5c displays the evolution of all calculated energy gaps in the system (Δ_h_^1^, Δ_e_^1^, Δ_e_^2^, Δ_e_^3^, Δ_e_^4^) for a range of twist angles θ between 0 and 1.8° as well as the energy gaps extracted from our devices via THz photocurrent measurements (Δ_h_^exp^, Δ_e_^exp^). In general, the calculated energy gaps are in good agreement with the values extracted from all our devices and measurements (Figures 3, 4, and Supporting Information Note 8), particularly given that any residual strain and/or twist-angle variations present in experimental samples will alter (reduce) the magnitude of gaps at the sDPs.^37^

**Figure 5:**
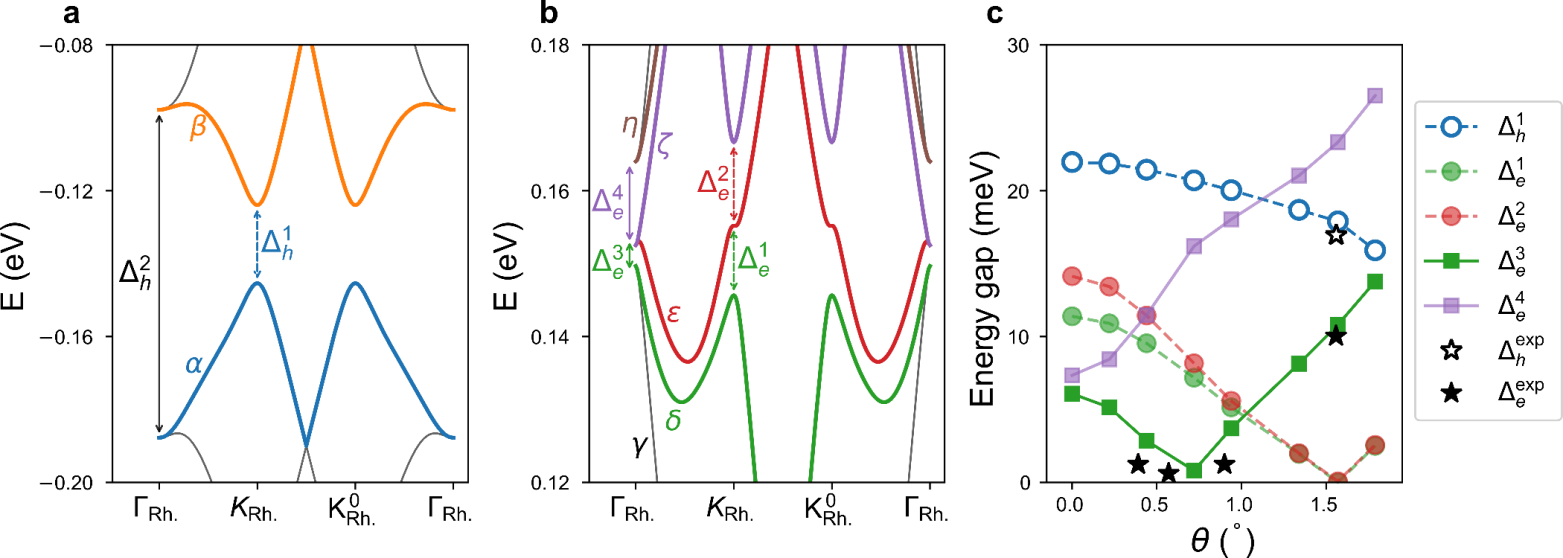
Band structure calculations of graphene/hBN moiré superlattice systems. (a) Calculated band structure of a graphene/hBN heterostructure, highlighting the existence of energy gaps Δ_h_^1^ at the hole-band sDPs with sizes around tens of meV. (b) Calculated band structure of a graphene/hBN heterostructure, highlighting the existence of energy gaps at the electron-band sDPs (marked Δ_e_^1^ to Δ_e_^4^) with sizes around a few meV. (c) Dependence of the energy gap sizes at the valence and conduction band sDPs on the misalignment angle θ. Experimental points (star symbols) represent the minimum energy gap values at the electron-band sDP (Δ_e_^exp^) or hole-band sDP (Δ_h_^exp^), extracted via THz photocurrent spectroscopy.

Moreover, we highlight that the evolution of the size of the local energy gaps in the conduction-band sDPs when increasing θ is nonmonotonic. This behavior is different from the simpler evolution of the size of energy gap in the valence-band sDP with θ (Δ_h_^1^ decreases for larger θ).

The existence of energy gaps at the sDPs with energy scales on the order of ∼meV promotes optical transitions at THz frequencies due to enhanced joint density of states (JDOS) related to the neighboring moiré minibands (see JDOS calculations in Supporting Information Note 9). These optical transitions are vertical (i.e., direct) and occur at specific ***k***-points in the reciprocal space, given the fact that photoexcitation processes satisfy momentum conservation and THz photons have a very small momentum with respect to the dimension of the Brillouin zone of the system. In consequence, as shown in Figure 6a,b, the interband optical conductivity of graphene moiré superlattices shows a notable spectral weight at doping levels *E*_F_ = ±*E*_sDP_, when the incoming energy of the THz radiation ℏω exceeds the distinct energy gap(s) present in the system (see annotations Δ_h_^1^, Δ_e_^1^, Δ_e_^2^, Δ_e_^3^, Δ_e_^4^ in the two panels, which correspond to the energy levels highlighted in Figure 5a,b). In more detail, the interband activity at the hole band sDP (i.e., doping level *E*_F_ = −*E*_sDP_, Figure 6a) acquires nonzero values at energies larger than Δ_h_^1^ and thus covers part of the THz energy range. In contrast, due to the presence of smaller gaps, the interband activity in samples with θ < 1° is appreciable in most of the THz range (i.e., for energies > Δ_e_^3^ ∼ 3 meV) at the conduction band sDP (doping level *E*_F_ = +*E*_sDP_, Figure 6b). All these trends are in close agreement with the overall photoresponse measured around the valence and conduction band sDPs at different THz frequencies in our devices (Figures 3, 4, and Supporting Information Note 8).

**Figure 6:**
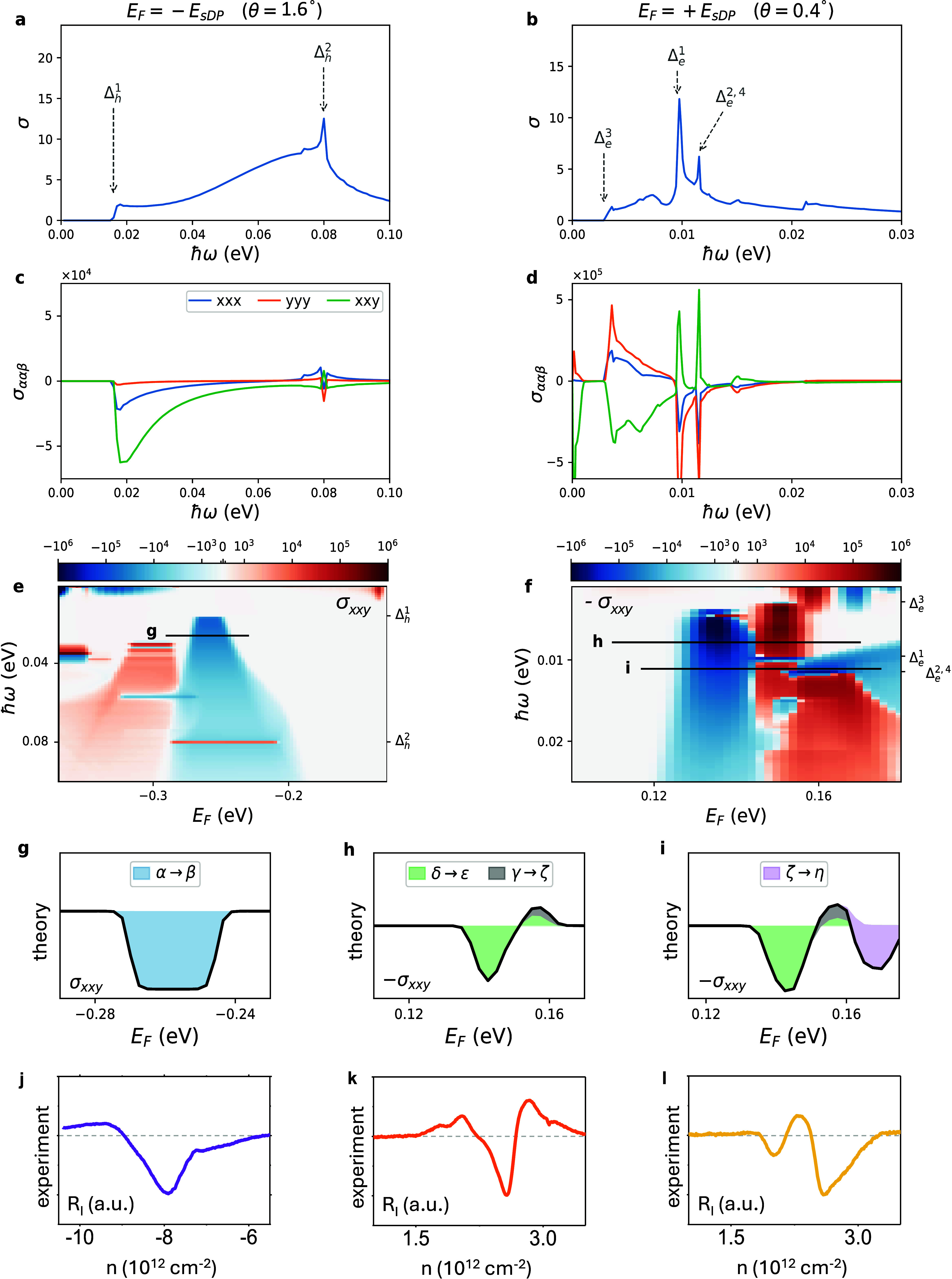
First- and second-order conductivity calculations of graphene/hBN moiré superlattice systems. Comparison with experiments*.* (a) Interband conductivity of graphene/hBN moiré superlattices as a function of the excitation energy ℏω at the position of the sDP in the valence band (doping level *E*_F_ = −*E*_sDP_) for a simulated sample with θ = 1.6°. (b) Interband conductivity of graphene/hBN moiré superlattices as a function of the excitation energy ℏω at the position of the sDPs in the conduction band (doping level *E*_F_ = +*E*_sDP_) for a simulated sample with θ = 0.4°. (c) Dependence of the shift conductivity components as a function of the excitation energy ℏω at the position of the sDP in the valence band (doping level *E*_F_ = −*E*_sDP_) for the simulated sample with θ = 1.6°. (d) Same with panel (c) for the conduction band (doping level *E*_F_ = +*E*_sDP_) and the simulated sample with θ = 0.4°. (e, f) Maps of the shift conductivity component σ_*xxy*_ as a function of the excitation energy ℏω and the doping level *E*_F_ around the position of the sDPs in the valence band, for the simulated sample with θ = 1.6°, panel (e), and in the conduction band, for the simulated sample with θ = 0.4°, panel (f). (g) Linecut of the calculated σ_*xxy*_(*E*_F_) in panel (e) around the valence band sDP for an excitation energy ℏω larger than Δ_h_^1^. (h, i) Linecuts of the calculated σ_*xxy*_(*E*_F_) in panel (f) around the conduction band sDPs for excitation energies ℏω larger than Δ_e_^3^ and Δ_e_^1^, respectively. (j) Measured *R_I_* of device A (θ ≈ 1.6°) near the valence band sDP for an excitation frequency of 4.1 THz. (k, l) Measured *R_I_* of device D (θ ≈ 0.4°) near the conduction band sDPs for excitation frequencies 0.3 and 0.6 THz, respectively. Dotted gray lines in panels (j–l) correspond to *R_I_* = 0.

#### Quantum Geometric Photocurrents

For completeness, we now consider the microscopic mechanism giving rise to a robust zero-source-drain bias photocurrent in our graphene moiré superlattice systems for excitation energies larger than the energy gaps present at the valence and conduction band sDPs. We remark that broken inversion symmetry and a local gap opening at an avoided crossing of Bloch bands induce a finite Berry curvature in the bands immediately above and below the energy gap. This geometric property is a consequence of Bragg scattering from the moiré superlattice, which mixes the pseudospin textures in the system.^39^ Furthermore, the peaked *R*_*I*_(*n*) observed at the doping levels ±*n*_SP_ cannot be explained by intraband activity, but it is more characteristic of intraband transitions. Given also the fact that the measured photoresponse arises from linearly polarized light, is highly sensitive to the atomic configuration of the graphene/hBN superlattice (i.e., to the misalignment angle θ) as well as the frequency of the incoming THz radiation, one can reasonably assign its origin to quantum geometric shift photocurrents occurring in vdW heterostructures with broken inversion symmetry.^17^ Such phenomena are second-order responses which arise as a result of a real-space displacement experienced by an electron wave function upon an optical transition and have been recently observed in moiré systems such as twisted bilayer graphene, around the main DP.^10,40^

The dependence of three components of the shift conductivity (σ_*xxx*_, σ_*yyy*_, σ_*xxy*_) with the excitation energy ℏω at the two selected chemical potentials in the valence and conduction minibands, *E*_F_ = −*E*_sDP_ and *E*_F_ = +*E*_sDP_, is shown in Figure 6c,d respectively. The induced photocurrent in a real system will contain several contributions, each proportional to a different shift conductivity component, with their relative weights dictated by the exact alignment of the device geometry and the polarization of the THz radiation.^17^ In general, all components of the shift conductivities become nonzero at excitation energies exceeding the energy gaps of the valence and conduction band sDPs (ℏω > Δ_h_, Δ_e_), with all three σ_*xxx*_, σ_*yyy*_, σ_*xxy*_ showing prominent peaks at these energies. σ_*xxy*_, which has the largest magnitude of the components shown, will now be used as a representative example to further analyze the shift photocurrent behavior near the valence and conduction band sDPs. Note that we may also invert its sign, which has the effect of reversing the arbitrary positive direction of one of the spatial directions, for an easier comparison with experimental results.

Figure 6e,f maps the evolution of σ_*xxy*_ as a function of *E*_F_ and ℏω close to the valence and conduction sDPs, respectively. Around the valence band sDP (Figure 6e), the largest shift current occurs when the chemical potential is placed within the energy gap (position *E*_F_ = −*E*_sDP_). Getting away from the sDP, σ_*xxy*_ diminishes and eventually vanishes due to Pauli blocking.^17^ An equivalent situation occurs in the vicinity of the conduction band sDP (Figure 6f). The main difference lies in the fact that, due to the more convoluted band structure existing at these energies (Figure 5b), the dependence of the calculated shift photocurrent on *E*_F_ may exhibit several sign changes and relative maxima at different excitation energies ℏω. This can be seen more clearly by examining horizontal cuts taken along the black lines in Figure 6e,f which are shown in panels (g–i). The minimum at the valence band sDP (Figure 6g) is relatively simple and can be exactly identified with transitions between bands α and β (Figure 5a and Figure S11), once the excitation energy is ℏω > Δ_h_^1^ (the minimum energy gap separating these bands). The conduction band cuts display a more complex behavior around *E*_F_ = +*E*_sDP_, as even contributions from the same two bands can have opposite signs. This is shown in Figure 6h for transitions between the bands labeled δ and ε in Figure 5b, where transitions at lower *E*_F_ (i.e., those at *K*_Rh._, requiring ℏω > Δ_e_^1^) have an opposite sign to those at slightly higher *E*_F_ (i.e., at Γ_Rh._, requiring ℏω > Δ_e_^3^). Increasing the excitation energy further allows transitions between other bands, as demonstrated in Figure 6i, where the additional contribution of transitions between bands ζ and η gives rise to an additional sign change and maxima. Intriguingly, as shown in Figure 6j–l, we observe similar complex interband photocurrent patterns in the measured photoresponse of some of our experimental devices. Specifically, the measured response in device A (θ ≈ 1.56°) at 4.1 THz (Figure 6j) shows a photocurrent *R*_*I*_(*n*) with a single wide minima occurring at a carrier density *n* ≈ −8 × 10^12^ cm^–2^ (i.e., Fermi level *E*_F_ ≈ −0.27 eV), which is indicative of a transition between two bands separated by a rather large gap existing at the valence band sDP. Both the line shape and the Fermi level position of the minimum agree well with the calculated σ_*xxy*_ (*E*_F_) for the simulated device with θ = 1.6° (Figure 6g). Meanwhile, multipeak structures occurring at Fermi levels between 0.14 and 0.16 eV are observed (Figure 6k,I) for device D (θ ≈ 0.4°) at 0.3 and 0.6 THz, respectively. Such features closely match the curves σ_*xxy*_ (*E*_F_) simulated for a corresponding device with θ = 0.4° (Figure 6h,i) and are thus indicative of the rich interband transition landscape provided by the various sub-bands present at the conduction band sDP.

#### Comparison with Other Techniques

The presented results prompt gate-tunable THz photocurrent spectroscopy as a robust and comprehensive technique to probe several features of the miniband electronic structure of graphene in the presence of moiré potentials, including Fermi surface parameters, marked energy levels, and subtle quantum geometry fingerprints of these electronic states. This is because the measured photoresponses show well-defined dependences (on both excitation energy and the chemical potential) when electrons transition within the same energy band or between the different bands present in these systems, and hence, the technique is sensitive to the distinct electronic features and band parameters.

An intraband-type photocurrent occurs at the lowest THz frequencies and depends on the details of the electronic Fermi surface of the system. In particular, as shown in the former sections, intraband photoresponses are sensitive to the overall DOS of the system and thus to relevant miniband parameters such the valley degeneracy *g*_*v*_^(DP)^ and the Fermi velocity *v*_F_^(sDP)^ of charge carriers at the satellite Dirac points. Interestingly, one can extract these parameters in a simple way from the recorded THz photoresponse *R*_*I*_(*n*), only by examining the ratio of the responsivity *R*_*I*_ measured around the main and satellite Dirac points δ*R*_*I*_^sDP^/δ*R*_*I*_^DP^ (eq 1). More conventional techniques reported in literature which are sensitive to *g*_*v*_^(DP)^ and *v*_F_^(sDP)^ such as scanning tunneling microscopy (STM)^1^ or capacitance spectroscopy^33^ measurements are able to disclose this information in a more convoluted way by monitoring (and fitting) the evolution of the DOS of the system with respect to the applied gate-voltage or device temperature, respectively.

Moreover, the lack of inversion symmetry existing in these moiré materials results in the presence of tiny (<20 meV) energy gaps at both valence and conduction bands sDPs as well as the generation of quantum geometric shift (bulk) photocurrents when the excitation energy of the incoming radiation is larger than the size of these energy gaps. The presence of this distinct type of interband photocurrent allows to probe key miniband parameters, including the actual size of the existing bandgap at the valence band sDP (Δ_h_) as well as the more complicated quantum texture present at the conduction band sDPs, featuring a number of avoided band crossings which give rise to a series of local energy gaps (Δ_e_) between consecutive bands (Figure 5b). Intriguingly, some of the extracted parameters (e.g., energy gaps Δ_e_) are undetectable by alternative techniques reported in the literature. The absence of a well-defined gap (i.e., a band gap) in the conduction band sDP (or equivalently, the existence of a Fermi surface and an intraband contribution^35^ at these energies) impedes the observation of a nonzero Δ_e_ via commonly used techniques based on electrical measurements, including temperature-dependent transport,^3^ tunneling spectroscopy,^20^ or capacitance spectroscopy^33^ (for example, see transport data and subsequent analysis of some of our devices in the Supporting Note 7). In addition, well-known optical techniques utilized to directly probe band structure of graphene-based superlattices, such as micro- or nanometer scale ARPES, have a limited energy resolution of ∼30 meV^22^ and thus are also not capable of resolving the tiny energy gaps <20 meV present at the conduction band sDPs.

We remark that gate-dependent THz photoresponses of an interband origin are not subjected to any of the two former limitations. On the other hand, photoexcitation processes occurring at the sDPs in electron- and hole-bands satisfy both momentum and energy conservation. For momentum conservation, the generation of electron–hole pairs is restricted to occur between states with the same wave vector value ***k*** in reciprocal space, owing to the very small momentum of the incident THz photons. In this sense, the measured bulk (interband) photocurrents are a direct consequence of vertical optical transitions that exclusively take place at specific ***k***-points in the vicinity of the sDPs and hence are extraordinarily sensitive to the size of energy gaps occurring in the mini bands of graphene-based moiré superlattice systems. Regions localized away from the sDPs, including ungapped regions as a result of, e.g., shunted edges,^40,41^ behave only as current collecting leads.

Finally, from a practical point of view, our measurements demonstrate that a conservative estimate of the energy resolution offered by gate-dependent THz photoresponse measurements is well below 1 meV. This is clearly shown in Figure 5c (main text) and in Figure S10c of Supporting Note 8, which exhibits clear interband transitions at radiation energies ∼0.6 meV (0.15 THz). Such a level of resolution is possible thanks to the notably distinct gate- and frequency-dependence exhibited by the different physical mechanisms giving rise to zero drain-bias THz photocurrents: whereas the optoelectronic response of interband origin (bulk photocurrents) is maximal at the sDPs, intraband photoresponses vanish at these energies. A detailed benchmarking between gate-dependent, THz photocurrent spectroscopy measurements and more conventional techniques to probe the different electronic parameters of graphene samples subjected to moiré superlattice potentials is depicted in Table 1.

**Table 1:** Comparison between Different Techniques Able to Probe Electronic Parameters of the Miniband Structure of Graphene/hBN Moiré Superlattices and Reported Energy Resolution

	miniband structure parameters and energy resolution				
probing technique	Δ_h_ [meV]	Δ_e_ [meV]	probe *g*_*v*_^(sDP)^?	probe *v*_F_^(sDP)^?	energy resolution [meV]
transport measurements (ref 3)	tens of meV (angle dependent)	NO^a^	NO	NO	∼1 to 2^c^
tunneling spectroscopy (ref 20)	tens of meV (angle dependent)	NO^a^	NO	NO	∼3
capacitance spectroscopy (ref 33)	NO^b^	NO^a^	YES	YES	∼5
STM (ref 2)	NO^b^	NO^a^	YES	YES	
ARPES (ref 27)	100 meV	NO^b^	NO	NO^b^	∼30
THz photocurrent spectroscopy (this work)	tens of meV (angle dependent)	0.6–12 meV (angle dependent)	YES	YES	<0.6^d^

a Parameter not reported in literature since the technique is sensitive to the overall bandgap of the system.

b Parameter not reported in literature due to the limited energy resolution offered by the technique.

c Uncertainty in the extracted gaps is set by the determination of the linear (thermally activated) regime for the fit.^2^

d Conservative value estimated from the minimum gap size demonstrated in the present work. This value is given by the minimum energy at which we observe an interband type of photocurrent in our samples (0.15 THz, see Figure S10c in Supporting Note 8). Measurement precision could eventually improve if additional low-energy lines would be available in the experimental setup.

## Conclusions

We have shown gate-tunable THz photocurrent spectroscopy as a robust and comprehensive technique to probe several features of the miniband electronic structure of graphene in the presence of moiré potentials, including Fermi surface parameters as well as intriguing energy levels and band textures not previously detected. On top of that, by observing bulk photocurrents in graphene moiré superlattices, our study demonstrates THz photocurrent measurements are also able to capture quantum geometric properties of electron states in the minibands of these materials (changes in the internal structure of electron wave functions between quantum states coupled by electromagnetic fields).

Finally, from a more technological perspective, we stress that the enhanced zero drain-bias responsivity values δ*R*_*I*_^sDP^/δ*R*_*I*_^DP^ appearing close to the sDPs in graphene/hBN moiré detectors with θ < 1° are subsequently accompanied by a lower noise equivalent power (NEP) at the satellite Dirac points NEP^sDP^ with respect to the main Dirac point NEP^DP^ (see Supporting Information Note 10). In particular, the NEP ratios NEP^sDP^/NEP^DP^ reach values down to 0.2 in these devices. As such, these two relevant device parameters (enhanced responsivity δ*R*_*I*_^sDP^/δ*R*_*I*_^DP^ and reduced NEP close to the satellite Dirac points) further demonstrate graphene/hBN moirè superlattices with alignment angles θ < 1° as convenient materials for sensitive and low noise THz detection.

## Methods

### Device Fabrication

Our aligned devices with angles between 0.4 and 1.6° are made of monolayer or bilayer graphene encapsulated between hBN flakes. Graphene and hBN flakes were first mechanically exfoliated and identified by using Raman spectroscopy. Bottom and top hBN flakes were chosen with similar thicknesses (between 20 and 30 nm) and identified via optical contrast and profilometer measurements. We chose elongated graphene and hBN flakes with straight edges to identify the crystallographic orientations of the graphene and hBN crystals. The stacking process of the moiré heterostructures was made using a dry transfer technique with a polypropylene carbonate film on a polydimethylsiloxane stamp. During the assembling process, we intentionally aligned close to 0° the straight edges of the top hBN and the MLG or BLG flakes (see further details in Supporting Information Note 1). The resulting stacked MLG/hBN moiré heterostructures were additionally characterized via Raman spectroscopy (see Supporting Information Note 3).

We then contacted all of our samples with one dimensional electrical contacts. For this, the heterostructures were patterned using electron beam lithography (EBL) to define contact areas using PMMA (6% in chlorobenzene) as resist. Subsequently, the heterostructures were dry-etched in an ICP-RIE Plasma Pro Cobra 100 in a SF_6_ atmosphere (40 sccm, pressure 6 mTorr, power 75 W at 10 °C), followed by an e-beam evaporation process at very low pressure (<5 × 10^–8^ Torr) of 3.5 nm of Cr and 65 nm of Au to deposit the metallic layers.

Afterward, two distinct processes were undertaken to shape and finalize our samples, depending on the device architecture. Top gates in SC and IDGT devices (samples A-E) are fabricated via EBL and e-beam evaporation of 5 nm Cr and 45 nm Au. Meanwhile, the definition of the MC geometry was undertaken via additional EBL and dry-etching processes.

### Electrical Measurements

Transport measurements were carried out via a standard lock-in technique where a pseudodc current (11.33 Hz) of 10 nA was injected into the drain and then collected in the source. The generated voltage drop was recorded by a lock-in amplifier (SR860). Measurements on devices A, B, D, and E were undertaken via a two-terminal configuration, while device C was characterized via a four-terminal configuration. The carrier density in the channel is controlled by the voltage applied either to the back-gates *V*_BG_ or top gates *V*_TG_ using a voltage generator (Keithley 2614B). For the back-gate bias, we directly applied a bias to the highly doped Si substrate with a typical range from −60 to 60 V (for SiO_2_ thickness of ∼300 nm). For the case of top-gate bias, values typically ranged from −4 to 4 V (for hBN thicknesses of ∼20 nm.

### THz Photoresponse Measurements

All of our graphene-based moiré superlattice devices were placed inside a cryostat system (ARS μDrift Nanoscience Cryostat) with optical access to perform the measurements. In our experiment, we collected the THz photocurrent through the source and drain electrodes at zero bias. For low frequencies, the measurements were performed using a THz source based on Schottky diode multiplier chains (TeraSchottky from Lytid) to generate output THz linear polarized frequencies at 0.075 THz (optical output power of ∼50 mW), 0.15 THz (optical output power of ∼30 mW), 0.3 THz (optical output power of ∼6 mW), and 0.6 THz (optical output power of ∼0.3 mW). For high frequencies, measurements were performed with a quantum cascade continuous-wave laser (TeraCascade 2000 series from Lytid) with output linearly polarized lines at 2.5 THz (optical output power of ∼3.2 mW), 2.9 THz (optical output power of ∼2.1 mW), 4.1 THz (optical output power of ∼3.2 mW), and 4.7 THz (optical output power of ∼4.8 mW). The output THz radiation, electrically modulated at 667 Hz, was collimated with an off-axis gold parabolic mirror and finally focused on the moiré THz devices by using a THz lens made of TPX (poly­(methyl pentene)) with a focal distance of 100 mm. Linearly polarized THz radiation was used to undertake all of the measurements. The photocurrent generated in the device channel was collected in the drain contact, amplified, and measured using a low noise current preamplifier (Stanford Research, SR570) in series with a lock-in amplifier (SR860). Carrier density on the channel was controlled by back or top gates using a voltage generator (Keithley 2614B). Source terminals were kept grounded during the photocurrent experiments.

### Tight Binding and Shift Conductivity Calculations

Periodic twisted graphene/hBN structures were first generated by finding superlattice vectors in each of the untwisted layers that had almost identical length and a relative rotation similar to the desired twisted angle. Periodic twisted graphene/hBN structures were first generated by finding two lattice vectors, one from each of the untwisted layers, that have almost identical lengths and a relative rotation similar to the desired twist angle. These two vectors were then perfectly aligned by rotating the hBN layer and applying a miniscule strain (of magnitude 0.01% or less), and the resultant vector gives one of the superlattice vectors of a rhombohedral unit cell, with the second superlattice vector given by a $\frac{\pi }{3}$ rotation.

The 0.44°-rotated system shown in the main text contains 10424 atoms in its unit cell, and its hBN layer is subject to a biaxial strain ε = 1.9 × 10^–5^. The electronic properties of the resulting twisted, multilayered graphene/hBN systems are described using a tight-binding Hamiltonian $\hat{H}=\sum_{i}\epsilon_{i}c_{i}^{†}c_{i}-t(\vec{d})\sum_{\left\langle i,j\right\rangle }(c_{i}^{†}c_{j}+c_{j}^{†}c_{i})$ , where *c*_*i*_^†^ and *c*_*i*_ are the creation and annihilation operators for an electron in the p_z_ orbital at site *i*. The onsite energies ϵ_*i*_ depend on the atomic species, with ϵ_C_ = 0.0 eV, ϵ_B_ = 3.34 eV and ϵ_N_ = −1.4 eV. The hopping parameter $t(\vec{d})$ depends on the distance vector between both sites and is given by $-t(\vec{d})=V_{\mathrm{pp}\pi }(d)\left[1-{\left(\frac{\vec{d}\cdot \vec{e_{z}}}{d}\right)}^{2}\right]+V_{\mathrm{pp}\sigma }(d){\left(\frac{\vec{d}\cdot \vec{e_{z}}}{d}\right)}^{2}$ , and its two components give both the in-plane and out-of-plane contributions to the hopping. The transfer integrals *V*_ppπ_(*d*) and *V*_ppσ_(*d*) can be written in terms of their (unstrained) graphene values as $V_{\mathrm{pp}\pi }(d)=V_{\mathrm{pp}\pi }^{0}e^{-d-a_{0}/r_{0}}$ and $V_{\mathrm{pp}\sigma }(d)=V_{\mathrm{pp}\sigma }^{0}e^{-d-d_{0}/r_{0}}$ , with *V*_ppπ_^0^ = −2.7 eV and *V*_ppσ_^0^ = 0.48 eV. *a*_0_ = 1.42 Å and *d*_0_ = 3.35 Å are the corresponding unstrained in-plane and out-of-plane atomic separations, *r*_0_ = 0.453 Å is a decay length, and $\vec{e_{z}}$ is the unit vector in the out-of-plane direction. We include all hopping terms whose corresponding separation in the xy-plane is less than a cutoff of 2.94 Å.

The band structures in Figure 5 are obtained by directly solving for the eigenvalues of the Bloch Hamiltonian. The optical and shift conductivities require the calculation of matrix elements^18,42,43^ of the form *h*_*ab*_^α^ = ⟨*a* | ∇_*k*_α__ *H* | *b* ⟩, where | *a* ⟩, | *b* ⟩ are eigenstates of the Hamiltonian and α = *x*, *y*, *z*. To calculate the optical conductivity, we follow the approach in ref 42, which averages the *x* and *y* components. We also employ the improved triangular method introduced in ref 42 to replace the integration over the irreducible Brillouin zone (IBZ) with a sum of contributions from individual triangular sections into which it is divided, as this approach typically requires a sparser sampling of k-space. The results for the 0.44°-twisted system use 2145 *k*-points in the IBZ.

We follow ref 18 in our simulation of the shift current response, which is described by a rank-three tensor σ_ααμ_, where α, μ = *x*, *y*, respectively, indicate the spatial components of the induced currents and electric fields. We do not consider α, μ = *z* in this work due to the in-plane polarization of the radiation but note it has been considered in other works.^43^ The σ_ααμ_ results shown in the main text are calculated following eqs 10 and 11 in ref 18, where the *k*-space integrations are once more performed using the improved triangular method from ref 42 The different pairwise contributions, shown in Figure 6g–i are calculated by considering individual terms from the summation in eq 10 of ref 18.

## Supplementary Material



## Notes

Additional fabrication details, additional devices, geometrical details, mobility, extraction of alignment angle between graphene and hBN in the devices, THz photoresponse in additional devices, evolution of THz photoresponse with temperature, estimation the enhanced responsivity ratio, assessment of energy gaps at the main and satellite DPs via transport measurements and via THz photocurrent spectroscopy, and additional calculations and calculation of Noise equivalent power (PDF)
