# Supplementary material for: Unveiling the Miniband Structure of Graphene Moiré Superlattices via Gate-Dependent Terahertz Photocurrent Spectroscopy
Source: ACS Nano. 2025 Jul 21;19(30):27338–50. doi: 10.1021/acsnano.5c05306 (PMC12333420; doi:10.1021/acsnano.5c05306)
Supplement: Supplementary file 1 [file nn5c05306_si_001.pdf]

Supporting Information for

"Unveiling the Miniband Structure of Graphene Moiré Superlattices via Gate-dependent Terahertz Photocurrent Spectroscopy"

Juan A. Delgado-Notario[1], Stephen R. Power[2], Wojciech Knap[3,4], Manuel Pino[5], Jin Luo Cheng[6], Daniel Vaquero[7], Takashi Taniguchi[8], Kenji Watanabe[8], Jesús E. Velázquez-Pérez[1], Yahya Moubarak Meziani[1], Pablo Alonso-González[9], José M. Caridad[1,10*]*

[1]Departamento de Física Aplicada, Universidad de Salamanca, 37008 Salamanca, Spain

[2]School of Physical Sciences, Dublin City University, Glasnevin, Dublin 9, Ireland

[3]CENTERA Labs, Institute of High Pressure Physics, Polish Academy of Sciences, Warsaw 01-142, Poland.

[4]Centre for Advanced Materials and Technologies CEZAMAT, Warsaw University of Technology, Warsaw 02-822, Poland.

[5]Departamento de Física Fundamental (IUFFyM) y GIR Nanotecnología, Universidad de Salamanca, 37008 Salamanca, Spain

[6]GPL Photonics Laboratory, State Key Laboratory of Luminescence and Applications, Changchun Institute of Optics, Fine Mechanics and Physics, Chinese Academy of Sciences, Changchun, Jilin 130033, People's Republic of China and University of Chinese Academy of Sciences, Beijing 100039, China

[7]Zernike Institute for Advanced Materials, University of Groningen, 9747 AG Groningen, The Netherlands

[8]Research Center for Electronic and Optical Materials, National Institute for Materials Science, Tsukuba305-0044, Japan

[9]Department of Physics, University of Oviedo, Oviedo 33006, Spain

[10]Unidad de Excelencia en Luz y Materia Estructurada (LUMES), Universidad de Salamanca, Salamanca 37008, Spain

* *emails:* juanandn@usal.es, jose.caridad@usal.es

Keywords: Terahertz, Graphene, Two Dimensional Materials, Moiré superlattices, Spectroscopy, Miniband structure

Note 1 – Additional fabrication details.

All our devices are fabricated on highly doped Si substrates (acting as back-gate) with 300 nm thermally growth SiO_2 on top. To assemble the aligned moiré heterostructures (rotation angle $\theta < 2^{\circ}$) we intentionally select elongated flakes with constant width and straight edges (**Figures S1 a** and **b**). This helps us to identify the crystallographic orientation of hBN and graphene crystals and therefore ensure a precise alignment between flakes in the heterostructure (**Figure S1 c**). We further highlight that the use of high temperatures (up to 180 ºC) during the encapsulation process facilitates both the fabrication of clean interfaces[1] and most importantly, the final crystallographic alignment close to 0° of graphene and hBN crystals.

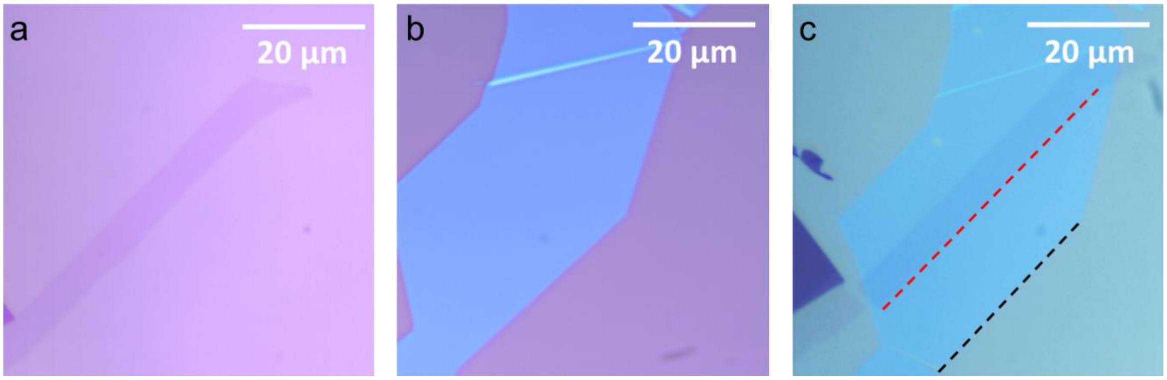

Figure S1. Stacking process of graphene moiré heterostructures. Optical image of the exfoliated flakes of **a,** Graphene, **b,** hBN and **c,** the fabricated half-heterostructure with the aligned top hBN and graphene flakes. Dashed lines indicate the crystallographic orientation of these crystals to guide the eye.

Table S1 shows specific details of all moiré superlattice devices fabricated in this work, including the use of monolayer (MLG) or bilayer (BLG) graphene

Device	Crystals	Top hBN Thickness (nm)	Bottom hBN Thickness (nm)	Device length L_{CH} (µm)
A	MLG and hBN	28	32	6
B	MLG and hBN	16	22	3
C	MLG and hBN	22	25	8.5
D	MLG and hBN	21	25	23.5
E	BLG and hBN	20	24	23.5

Table S1. Details of all graphene based heterostructures used in this work.

Note 2 – Additional graphene moiré THz devices, Device characterization and geometrical details.

Figure S2 depicts the transport characteristics of devices C, D and E studied in this work.

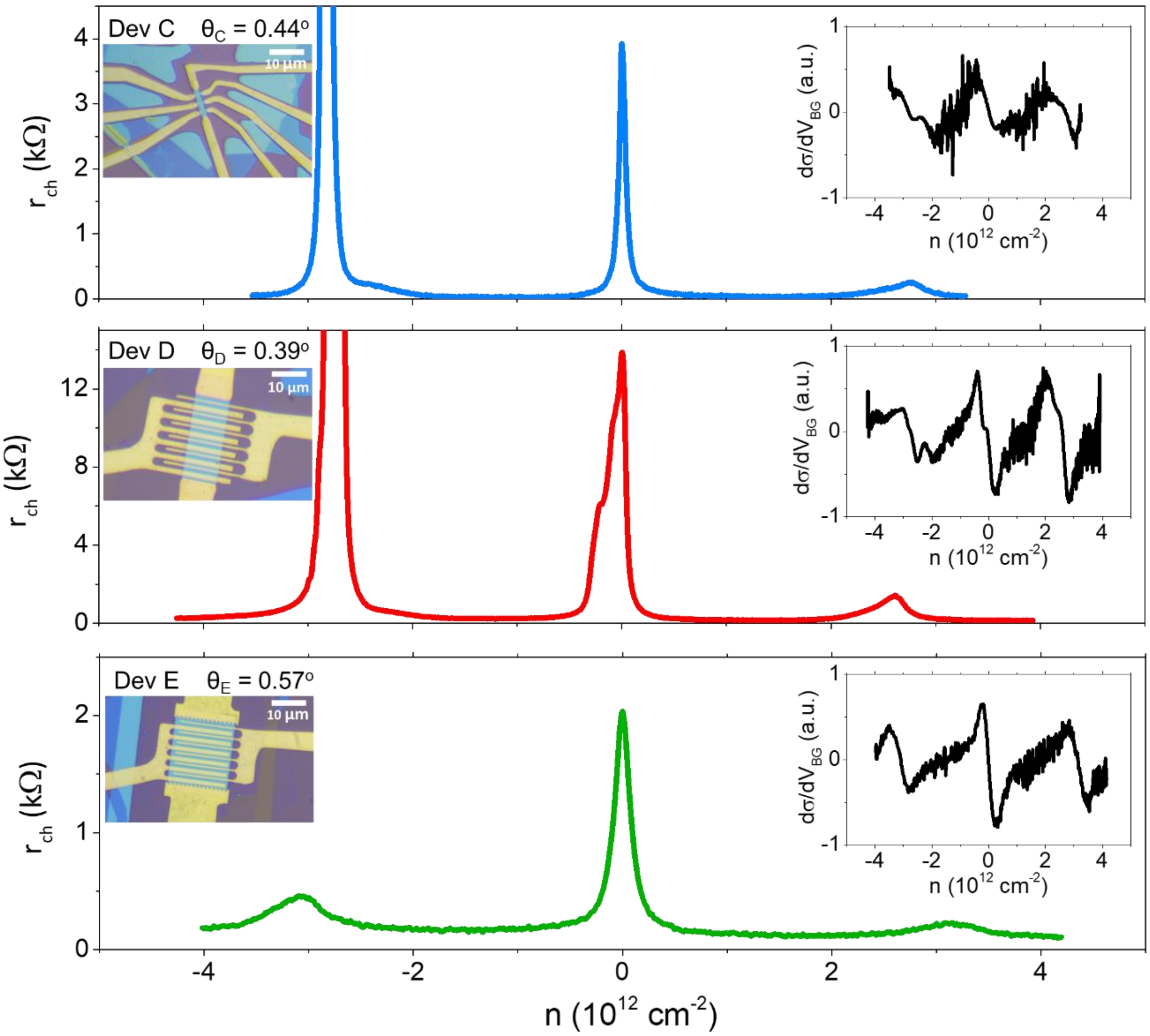

Figure S2: Electrical characteristics of additional graphene moiré devices C-E. Measured channel resistance, r_{ch}, as a function of the carrier density, n, for the three additional devices of the study at 10K. From top to bottom panels: device C (multiple-cross MLG/hBN photodetector with a θ = 0.44˚), device D (interdigitated dual-grating gate MLG/hBN device with a θ = 0.39˚), device E (interdigitated dual-grating gate BLG/hBN device with a θ =0.57˚). Left insets in each panel show the corresponding optical image of the moiré THz device. Right insets in each panel show the variation of the channel conductivity σ w.r.t. the gate voltage, $d\sigma/dV_G$ as a function of the normalized back-gate voltage.

In addition, detailed information about the geometrical parameters of all three device architectures fabricated for this study are highlighted in **Figure S3**.

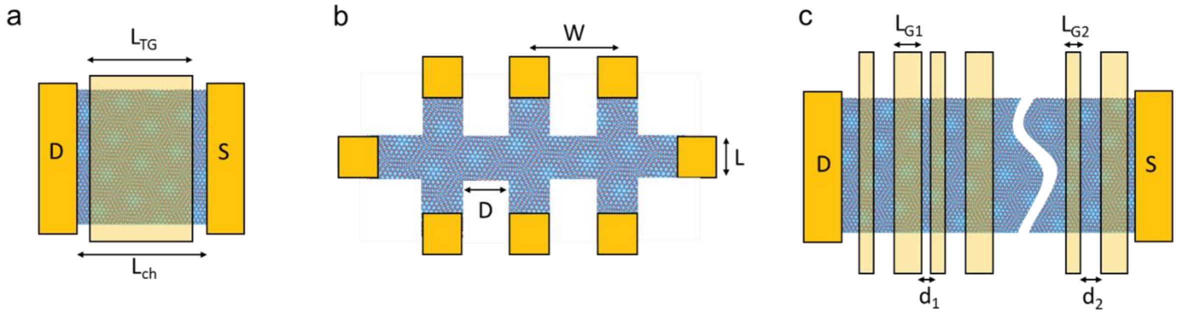

Figure S3. Schematic views of the fabricated moiré graphene-based superlattices devices. a, Schematic top view of the Short-Channel (SC) architecture (devices A and B). Dimensions are L_{ch} = 6 (3) µm and L_{TG} = 4.8 (2.5) µm for the device A (B). **b**, Schematic top view of the multicross (MC) bar geometry (device C). Dimensions are W = 2.5 µm and D = L = 1 µm. **c**, Schematic top view of the Ratchet type architecture of the Devices D and E. Top gates are characterized by an unit cell with a period of L = 3.75 µm (L_{G1} = 1.5 µm, L_{G2} = 0.75 µm, d_1 = 0.5 µm, d_2 = 1 µm). The unit cell was repeated 6 times.

Note 3 – Mobility of the measured devices.

The resistance of the device channel r_{ch}, the contact resistance r_c and mobility values μ for each device have been calculated from the measured resistance, using the procedure described by Ref. 2 and taking into account that the carrier density value n is given by the expression $n = C_c(V_{BG} - V_{DP})/e$, where C_c is the capacitance of the device, e is the electron charge, V_{BG} is the applied back-gate potential and V_{DP} is the gate-potential at which the resistance maxima is obtained (indicating the position of the main Dirac point). In device C, r_c was set to zero as its electrical characteristics were measured in a four-terminal configuration. The dc channel conductivity is extracted using the formula $\sigma = (L/W)/(r_{ch} - r_c)$, where L is the channel length, W is the channel width.

Figure S4 shows the obtained carrier mobility values, $\mu(n)$, for all our devices at T=10K. We consistently observe carrier mobilities reaching values higher than 10 m^2/Vs for holes and electrons in all our samples (see further details in **Table S2**).

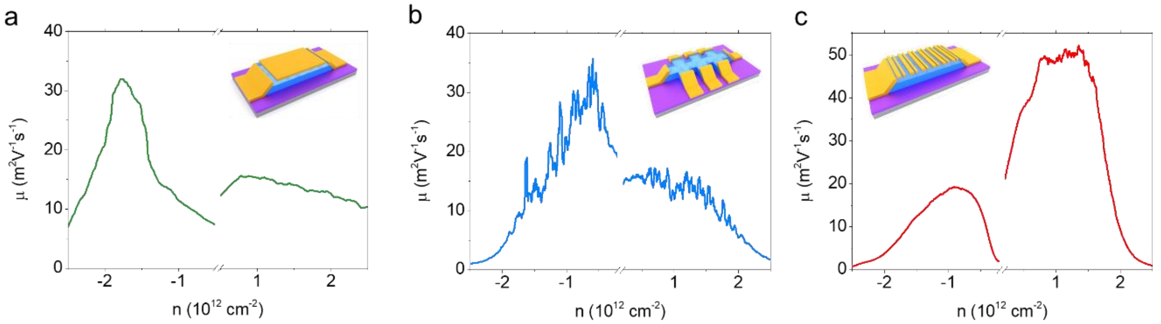

Figure S4. Carrier mobility in our devices at T=10K. Carrier mobility as a function of the carrier density for **a,** short channel device (device A), **b,** multi-cross device (device C) and **c,** interdigitated dual-grating gate device (device D). Insets in the panel show the corresponding architecture of the devices.

	V_{DP} (V)	r_c (Ω)	$\mu_{e,max}$ (m^2/Vs)	$\mu_{h,max}$ (m^2/Vs)
Device A	0.9	380	15.7	32.1
Device B	0.3	580	11.17	12.91
Device C	1.8	-	16.3	35
Device D	2.4	125	51	19.8
Device E	-1.3	67	24	18

Table S2. Electrical parameters at 10K. Gate-voltage at which the resistance maxima occur in the sample V_{DP}, contact resistance r_c, and maximum carrier mobility measured in the device at different densities for electrons $\mu_{e,max}$ and holes $\mu_{h,max}$ charge carriers.

Note 4 – Extraction of alignment angle between graphene and hBN in the devices used for this work.

Table S3 summarizes the moiré wavelength, λ_M, and twist angle, θ, extracted in our devices from transport and Raman spectroscopy measurements. Values obtained from both techniques are consistent, with the largest disagreement between average twist angles extracted from both type of measurements being 0.06 degrees.

	Raman spectroscopy		Transport measurements	
	λ_M (nm)	θ (degrees)	λ_M (nm)	θ (degrees)
Device A	7.6	1.56	7.4	1.62
Device B	10.27	0.93	10.4	0.9
Device C	12.84	0.42	12.75	0.44
Device D	12.96	0.4	12.98	0.39
Device E	-	-	12.15	0.57

Table S3. Extracted alignment parameters from Raman spectroscopy and transport measurements

A. via Raman measurements

Raman spectroscopy measurements were carried out at room temperature in air using a 532 nm laser with an incident power of 1 mW. The relative rotation angle θ and the moiré wavelength of graphene superlattices λ_M made of monolayer graphene shown in **Table S3** are extracted from the full width at half maximum ($FWHM$) of the measured Raman 2D peak, which is given by[3]

$$FWHM \approx 5 + 2.6\lambda_M \qquad \textbf{Eq. S1}$$

Moreover, the relation between λ_M and θ is:

$$\lambda_M = \frac{(1+\xi)a}{\sqrt{2(1+\xi)(1-cos\theta)+\xi^2}} \qquad \textbf{Eq. S2}$$

where a is the graphene lattice constant and ξ is the lattice mismatch between the graphene and the hBN lattices. **Figure S5** shows the measured 2D peak for devices A-D as well as the gaussian fits to these data used to extract the FWHM values. Specifically, we have obtained FWHM values of 24.77 cm^{-1} for device A, 31.7 cm^{-1} for device B, 38.4 cm^{-1} for device C and 38.7 cm^{-1} for device D. We further note that samples with relatively uniform twist angle are selected for this study. To do so, we measure the FWHM of the 2D peak[3,4] in five different positions per sample, verifying that such values are the same within the experimental error of our Raman spectrometer (±2cm^{-1}).

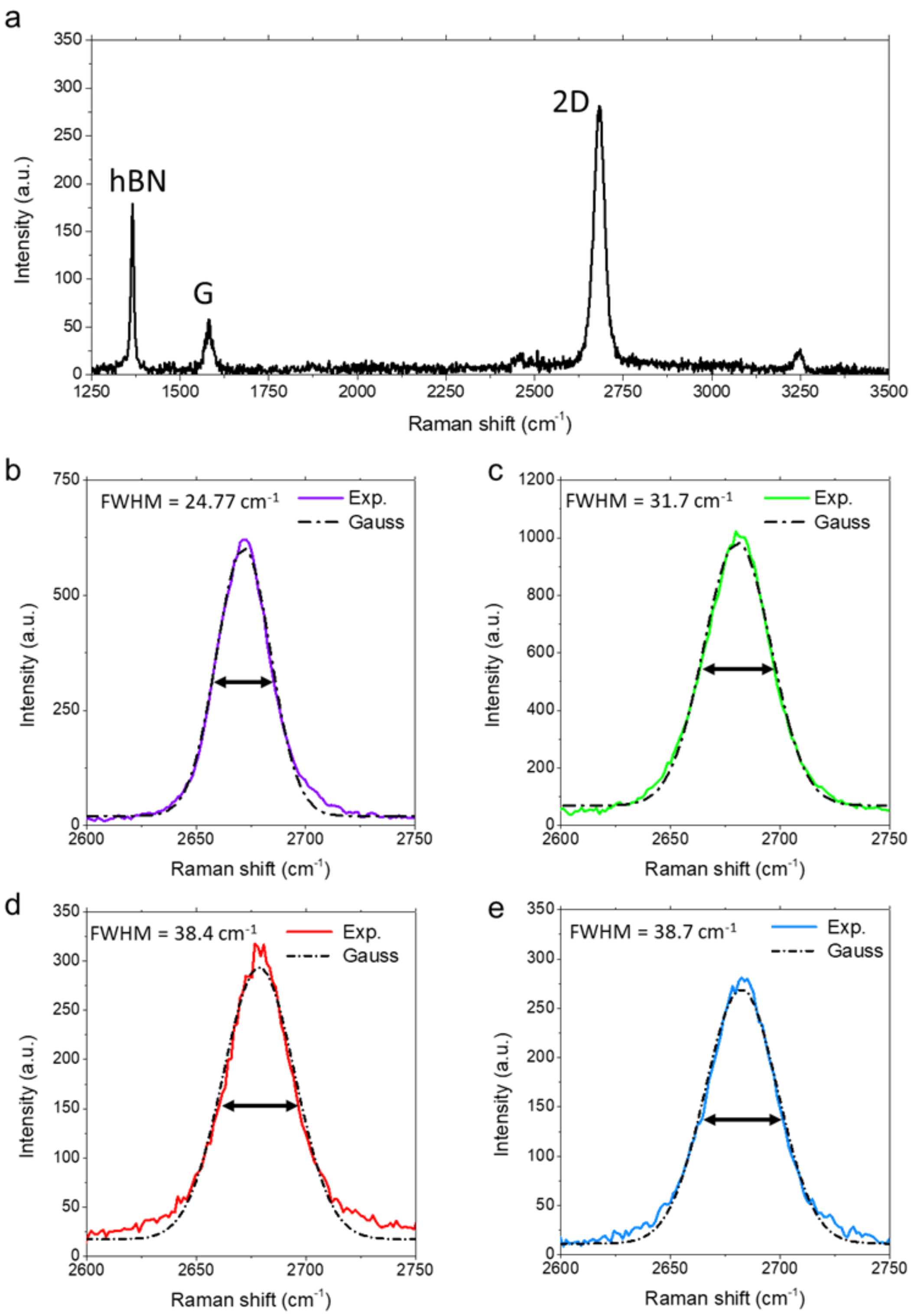

Figure S5: Raman spectra of the moiré graphene heterostructures. **a,** Typical Raman spectra of an aligned hBN/graphene superlattice. **b-e,** Gauss fitting of the 2D peak for the devices A, B, C and D respectively highlighting the value of the full width at half-maximum ($FWHM$).

B. via transport measurements

One can obtain λ_M and θ via transport measurements, taking into account[5] Eq. S2 and the fact that the resistance peaks associated to the satellite Dirac points occur at a charge density $n_{sp} = 4/A$, where $A = \sqrt{3\lambda_M^2}/2$ is the size of the moiré unit cell and the pre-factor 4 considers the spin and valley degeneracies in graphene.

From the gate potential $|V_{BG}-V_{DP}|$ at which the satellite points occur, n_{sp} in our devices is estimated to be n_{sp} is $8.4\cdot10^{12}$ cm^{-2} for device A, $4.27\cdot10^{12}$ cm^{-2} for device B, $2.84\cdot10^{12}$ cm^{-2} for device C, $2.74\cdot10^{12}$ cm^{-2} for device D and $3.3\cdot10^{12}$ cm^{-2} for device E.

Note 5 – THz photoresponse in our devices: additional data and analysis

Additional responsivity data

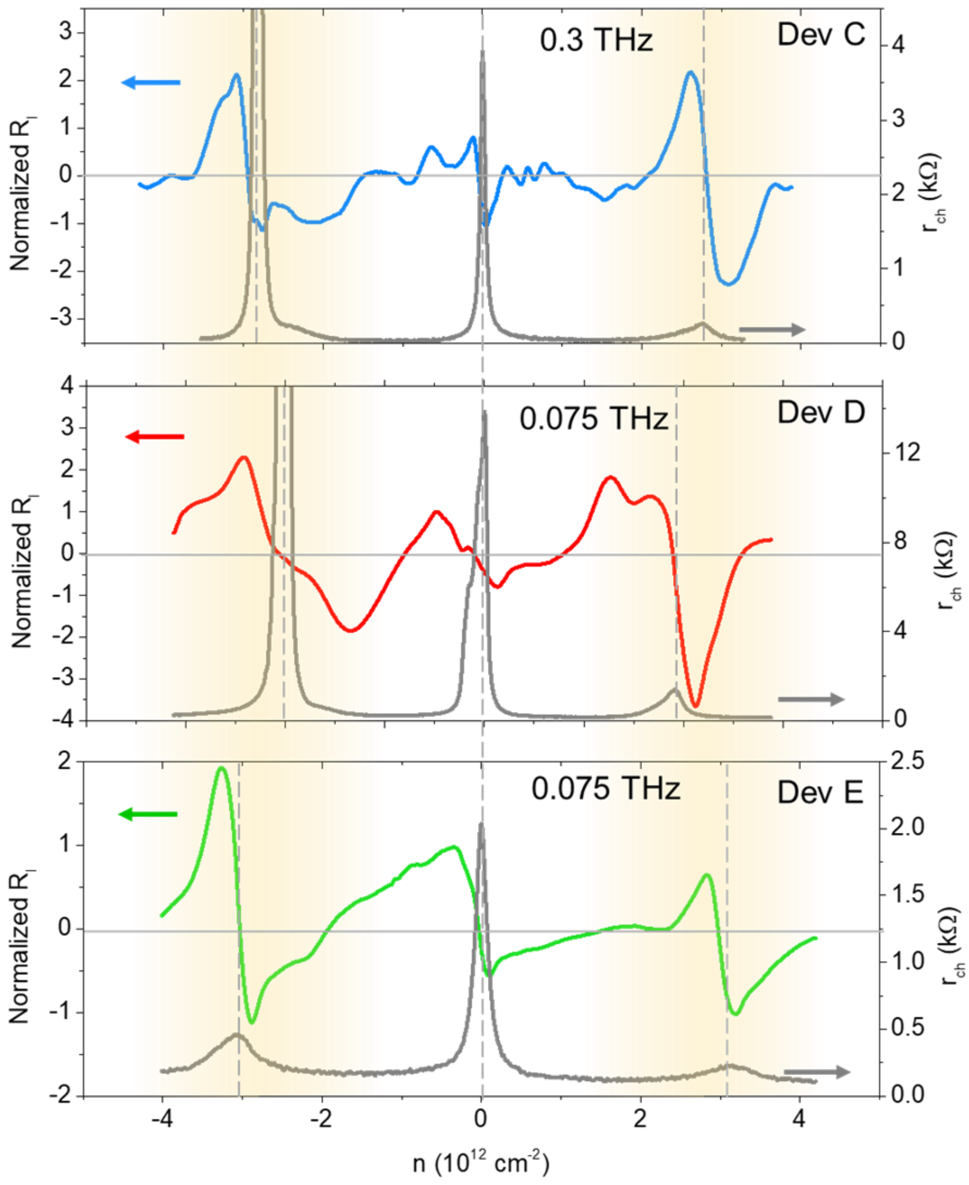

Figure S6. Low temperature photocurrent measurements. (Left axis) Normalized Photocurrent responsivity, R_I and (right axis) measured channel resistance, r_{ch}, as a function of the carrier density, n, for the Devices C (hBN/MLG/hBN), D (hBN/MLG/hBN) and E (hBN/BLG/hBN) under excitation of THz radiation. The temperature of the measurement is T = 10 K in all the panels.

Responsivity ratios for all our devices at 10K

Table S4 presents the comparison between the THz responsivities around the two sDPs ($\delta R_I^{sDP,h}$ or $\delta R_I^{sDP,e}$) with respect to the responsivity close to the main DP (δR_I^{DP}), in all moiré graphene/hBN photodetectors measured in this study. Such ratios are shown for two excitation frequencies (0.15 THz and 0.3 THz). The data show consistently responsivity ratios above unity in devices with twist angles smaller than 1 degree.

	0.15 THz		0.3 THz	
Device (twist angle)	$\delta R_I^{sDP,h}/\delta R_I^{DP}$	$\delta R_I^{sDP,e}/\delta R_I^{DP}$	$\delta R_I^{sDP,h}/\delta R_I^{DP}$	$\delta R_I^{sDP,e}/\delta R_I^{DP}$
A (1.62°)	1.06	0.72	0.63	0.2
B (0.9°)	1.75	3.4	1.26	2*
C (0.44°)	-	-	1.76	2.40
D (0.39°)	1.76	3.48	2.28	2.20*
E (0.57°)	1.83	1.29*	4.91	2.91*

Table S4. Enhancement factor for all studied devices at 10 K. Note that, whereas a photocurrent of an intraband origin always occurs at the valence band sDP, an intraband photocurrent may take place in the electron band sDP (cases marked with an asterisk ′*′ in the table).

Evolution of $R_I(n)$ with temperature

Figure S7 shows the evolution of the responsivity ratio (or enhancement factor) with temperature for our aligned graphene/hBN devices (twist angles θ<1°, devices B, C, D and E) at 0.3 THz. Here, we focus our attention on the ratio between the valence band sDP and the main DP ($\delta R_I^{sDP,h}/\delta R_I^{DP}$), where the measured photocurrent is exclusively of an intraband origin.

Overall, $\delta R_I^{sDP,h}/\delta R_I^{DP}$ decreases when increasing the temperature and shows enhancement factors >1 for temperatures *T* below 80-120K in all moiré devices, irrespectively if they are made of monolayer (Figure S7a) or bilayer (Figure S7b) graphene. This is a general behavior which can be understood by the presence of electron–electron Umklapp scattering as dominant scattering mechanism close to the sDPs in graphene-based moiré superlattices at high temperatures[6].

As an additional comment, we also observe that $\delta R_I^{sDP,h}/\delta R_I^{DP}$ remains > 1 up to 120K for the moiré device made from bilayer graphene (all studied monolayer graphene devices present ratios $\delta R_I^{sDP,h}/\delta R_I^{DP}$ >1 for temperatures <100K). Such trend may be related to the fact that, due to the presence of additional intrinsic scattering sources including shear phonon scattering, the

responsivity δR_I^{DP} decreases more rapidly in samples made of bilayer graphene than in those made of monolayer graphene when increasing the temperature[7].

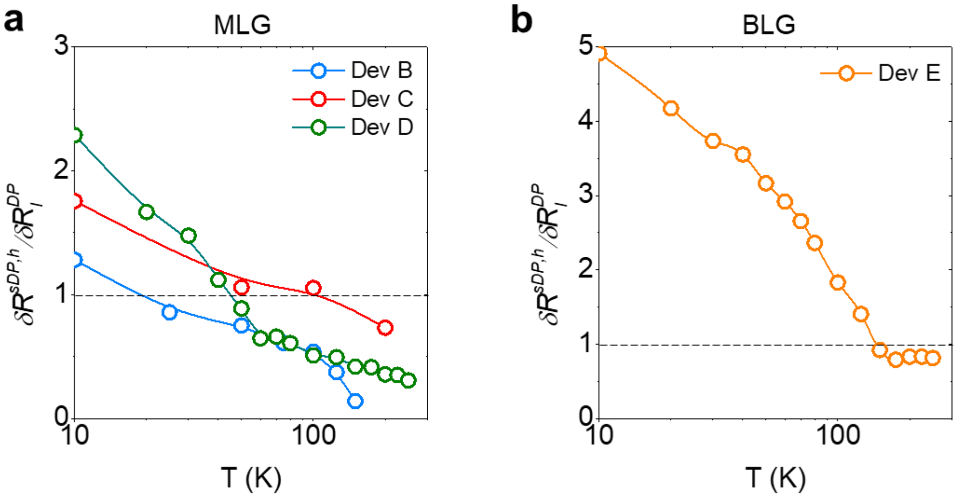

Figure S7: Evolution of the responsivity ratio $\delta R_I^{sDP,h}/\delta R_I^{DP}$ in our aligned devices (θ<1°) at 0.3 THz. $\delta R_I^{sDP,h}/\delta R_I^{DP}$ is plotted as a function of temperature for aligned (a) monolayer devices B, C, D and (b) bilayer device E. In all graphs, experimental data points are connected by lines to guide the eye.

Note 6 – Estimation the enhanced responsivity ratio $\boldsymbol{\delta R_I^{sDP}/\delta R_I^{DP}}$.

The broadband photoresponse of photodevices governed by intraband transitions follows the expression[8-11]:

$$R_I = -\frac{\delta U^2}{4P}\frac{d\sigma}{dV_{BG}} \qquad \textbf{Eq. S3}$$

where δU is the amplitude of the ac potential induced by the THz radiation in the device channel, P is the incident radiation power and σ is the dc conductivity of the material.

In order to estimate the ratio $\delta R_I^{sDP}/\delta R_I^{DP}$, we assess the ratios of the two main terms of the photocurrent responsivity in Eq.S3: δU and $d\sigma/dV_{BG}$.

<u>Estimation of term $d\sigma/dV_{BG}$</u>

First, we note the equivalence between $d\sigma/dV_{BG}$ and $d\sigma/dn$ in our devices is given by $d\sigma/dV_{BG} = \frac{C_c}{e} d\sigma/dn$, since the relation $n(V_{BG})$ in devices with a notable dielectric thickness > 20 nm is primarily dictated by the classical capacitance C_c[12] (i.e. the quantum capacitance contribution is not relevant in our devices).

In the diffusive transport regime, the dc conductivity in monolayer graphene is given by the Einstein relation[13]:

$$\sigma = e^2 D(E_F) v_F^2 \tau/2 \qquad \textbf{Eq. S4}$$

where $D(E_F) = (\sqrt{g_s g_v n})/(\sqrt{\pi}\hbar v_F)$ is the density of states at the Fermi level E_F, v_F is the Fermi velocity of charge carriers, τ is the carrier density dependent scattering time, and g_s, g_v are the spin and valley degeneracies of graphene, respectively.

In a diffusive system, the two main scattering events in the system can have a long- or short-range origin, and depend on n as $\tau \propto \sqrt{n}$ and $\tau \propto 1/\sqrt{n}$, respectively. These two mechanisms give rise to two contributions to the total conductivity σ (see Eq.S5), which are linear or independent on the carrier density n for long (σ_l) and short-range (σ_s) scattering events, respectively.

$$\frac{1}{\sigma(n)} = \frac{1}{\sigma_l(n)} + \frac{1}{\sigma_s} \qquad \textbf{Eq. S5}$$

We remark that the two conductivity contributions σ_l , σ_s encompass different scattering mechanisms that may occur in any graphene device[14-16].

In the case of graphene/hBN moiré superlattice devices, their total conductivity $\sigma^{SL}(n)$ can be estimated by considering three contributions σ, one from the main DP and two form the satellite DPs as[17]:

$$\frac{1}{\sigma^{SL}(n)} = \frac{1}{\sigma(n)} + \frac{1}{\sigma(n+n_{sDP})} + \frac{1}{\sigma(n-n_{sDP})} \qquad \textbf{Eq. S6}$$

Figure S8 shows the normalized estimated conductivity $\sigma^{SL}(n)$ and its variation with respect to the carrier density of the channel $d\sigma^{SL}/dn$.

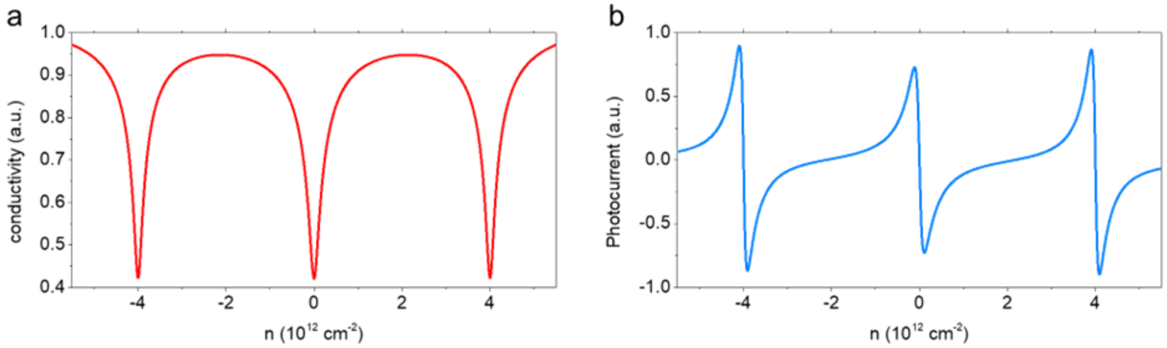

Figure S8. a, Estimated dependence of the conductivity with n in graphene moiré superlattice devices σ^{SL} calculated from Eq.S6 and **b,** photocurrent calculation via $d\sigma^{SL}/dn$.

Importantly, $d\sigma^{SL}/dn$ shows a similar magnitude of the photoresponse close to the main and satellite Dirac points, which would result in a responsivity ratio $\delta R_I^{SP}/\delta R_I^{DP}$ close to unity. More explicitly, the estimated responsivity ratio from this factor is roughly given by:

$$\left(\frac{d\sigma}{dn}\right)^{(sDP)} / \left(\frac{d\sigma}{dn}\right)^{(DP)} \approx \left(\sqrt{g_v^{(sDP)}}\, v_F^{(sDP)}\right)/\left(\sqrt{g_v^{(DP)}}\, v_F^{(DP)}\right) \qquad \textbf{Eq. S7}$$

In the former expression, $v_F^{(DP)}, v_F^{(sDP)}$ and $g_v^{(DP)}$, $g_v^{(sDP)}$ are the Fermi velocities and valley degeneracies of graphene carriers close to the main and satellite Dirac points, respectively. The maximum ratio $\delta R_I^{SP}/\delta R_I^{DP}$ that can be estimated from Eq. S7 is ~1.26 for the case $v_F^{(sDP)} \sim 0.73\, v_F^{(DP)}$ and $g_v^{(sDP)} = 3g_v^{(DP)}$ (maximum values reported in literature for these electronic parameters[18,19]). Such ratio is, therefore, close to 1 and notably smaller than the ones observed experimentally in our devices with θ <1°. As such, the term $d\sigma/dV_{BG}$ alone cannot explain the observed enhancement of the measured photocurrent near the satellite Dirac points.

AC potential induced in the channel by the THz radiation, δU

The term δU is commonly approximated in literature[8-11] by the constant value U_a, amplitude of the ac potential between the lobes of the antenna. Nonetheless, in practical devices, δU depends on the coupling channel material. In this sense, we note that δU can be related to the carrier density oscillation in the material δn via[20] $\delta U = (e^2/C)\delta n$. In the former expression, C is the dynamic capacitance and δn can be approximated to the Fermi level E_F and the equilibrium carrier density of the channel n_{eq} as[21] $\delta n = (\partial n_{eq}/\partial E_F)\partial E_F$. Moreover, to a first approximation (zero

temperature and zero carrier density), the compressibility $\partial n_{eq}/\partial E_F$ is given by the density of states at the Fermi level of the system $D(E_F)$.
In this sense, δn is different at Fermi levels close to the main and satellite Dirac points in a graphene moiré superlattice device. In consequence, the ratio $\delta R_I^{sDP}/\delta R_I^{DP}$ can be estimated as:

$$\frac{\delta U^{2(sDP)}}{\delta U^{2(DP)}} = \frac{\left(D(E_F)^{(sDP)}\right)^2}{\left(D(E_F)^{(DP)}\right)^2} = \frac{g_v^{(sDP)}\left(v_F^{(DP)}\right)^2}{g_v^{(DP)}\left(v_F^{(sDP)}\right)^2} \qquad \textbf{Eq. S8}$$

By inserting Eq. S7 and S8 in Eq. S3, we have

$$\frac{\delta R_I^{sDP}}{\delta R_I^{DP}} \approx \frac{\left(g_v^{(sDP)}\right)^{3/2} v_F^{(DP)}}{\left(g_v^{(DP)}\right)^{3/2} v_F^{(sDP)}} \qquad \textbf{Eq. S9}$$

which is Eq.1 in the main text.

Note 7 – Assessment of energy gaps at the main and satellite DPs via transport measurements

In this note, we assess the size of the energy gaps at the main and satellite Dirac points in one of our devices via temperature dependent transport measurements. This is a conventional technique used in literature to extract the energy gap of graphene moiré superlattice systems (see e.g. Ref 4). **Figure S9a** shows the evolution of $r_{ch}(n)$ at three different temperatures from 10K to 250K for device D ($\theta \sim 0.4$ degrees). The resistance maxima r_{ch}^{max} gets larger at lower temperatures at the hole band SDP and the main DP. Arrhenius plots showing the dependence of the minimum conductance of the device ($1/r_{ch}^{max}$) with *1/T* at the hole band SDP and main DP are depicted in **Figures S9b** and **S9c**, respectively.

From these data, the extracted energy gaps at these two DPs are $\Delta_h \sim 21$ meV and $\Delta \sim 23$ meV, respectively, values which are in good agreement with those probed in literature in similar samples via transport measurements[5]. More importantly, the values of Δ_h extracted via the here proposed THz photocurrent spectroscopy (**Figure 3** in the main text) are in excellent agreement with those extracted via transport measurements (**Figure S9b**). In contrast, r_{ch}^{max} is approximately constant at the conduction band SDP for the measured temperature range (it varies between 0.6 to 0.7 kΩ), behaviour which is also observed in additional graphene moiré superlattices reported in literature[22]. The fact that r_{ch}^{max} does not increase monotonically when lowering the temperature as expected for thermally activated transport is a direct consequence of the lack of an overall bandgap present around the satellite Dirac point in the conduction band (see calculated bandstructure in **Figure 5b** of the main text).

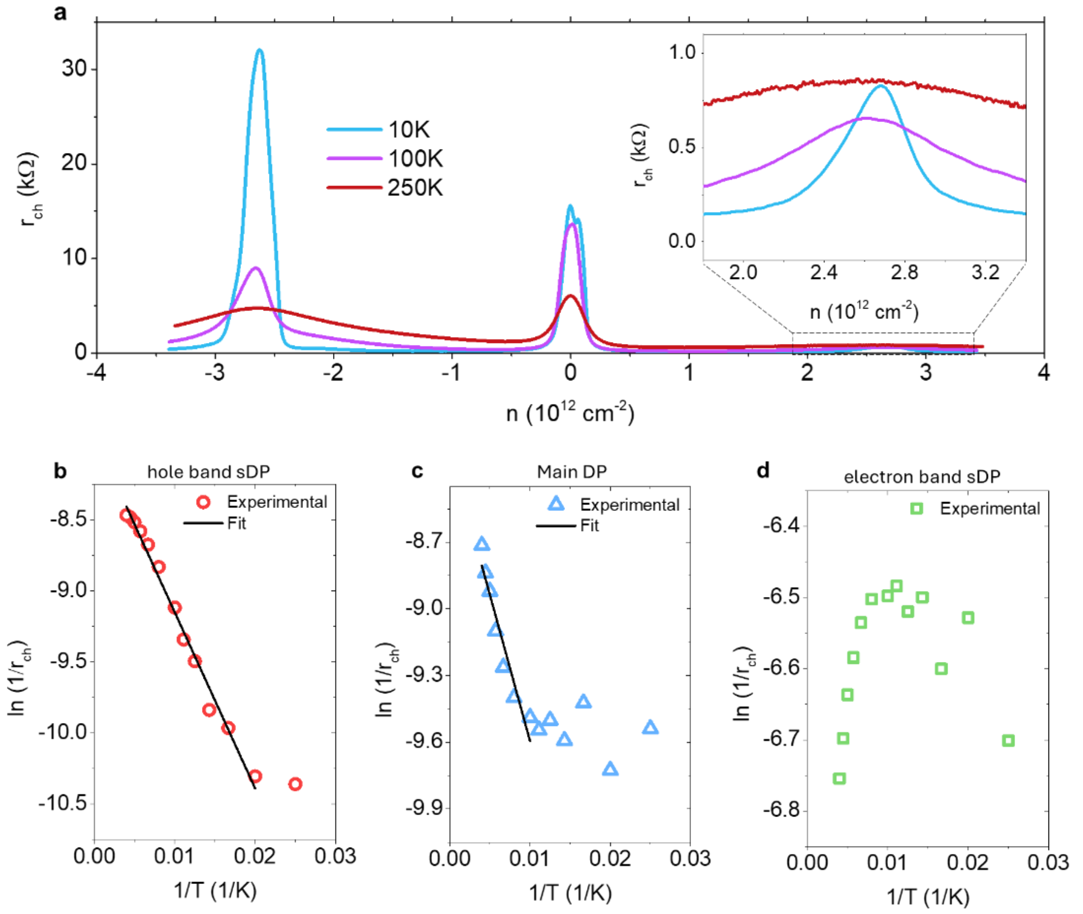

Figure S9: Evolution of channel resistance r_{ch} with temperature for device D. a, $r_{ch}(n)$ at three selected temperatures. **b,** Value of the minimum conductance ($1/r_{ch}^{max}$, with r_{ch}^{max} being the resistance maximum) measured in the channel close to the hole band sDP. The black line corresponds to a fit of the minimum conductance assuming thermal activated transport $1/r_{ch}^{max} \propto \exp(-\Delta_h/2K_BT)$, with $\Delta_h = 21$ meV. **c,** Value of the minimum conductance ($1/r_{ch}^{max}$, with r_{ch}^{max}) measured in the channel close to the main DP. The black line corresponds to a fit of the minimum conductivity assuming thermal activated transport $1/r_{ch}^{max} \propto \exp(-\Delta/2K_BT)$, with $\Delta = 23$ meV. **d,** Value of the minimum conductance ($1/r_{ch}^{max}$, with r_{ch}^{max} being the resistance maximum) measured in the channel close to the conduction band sDP.

Note 8 – Assessment of energy gaps at the electron-band SDP via THz photocurrent spectroscopy.

Figure S10 shows the responsivity $R_I(n)$ of different devices measured close to the electron-band sDP at different sub-THz frequencies (*f* = 0.075, 0.15, 0.3 and 0.6, respectively). Here, we can observe how $R_I(n)$ shows an intra-band photocurrent at all measured frequencies in devices with a large rotation angle (θ>1˚) between the graphene and hBN crystals (device A, see **Figure S10a**).

In contrast, devices with a lower rotation angle (θ<1˚) behave in a different way. Whereas $R_I(n)$ of devices D and E (see **Figure S10 b** and **c**) also show an intraband type of photoresponse at the lowest frequency (0.075THz); the lineshape of $R_I(n)$ evolves towards an interband-type of photorresponse at larger frequencies, above 0.3 THz and 0.15THz for devices D and E respectively.

From these measurements, we extract an approximate size of the energy gap to be Δ_e ≈1.2 meV and Δ_e ≈0.6 meV for devices D (θ~0.39˚) and E (θ~0.57˚), respectively.

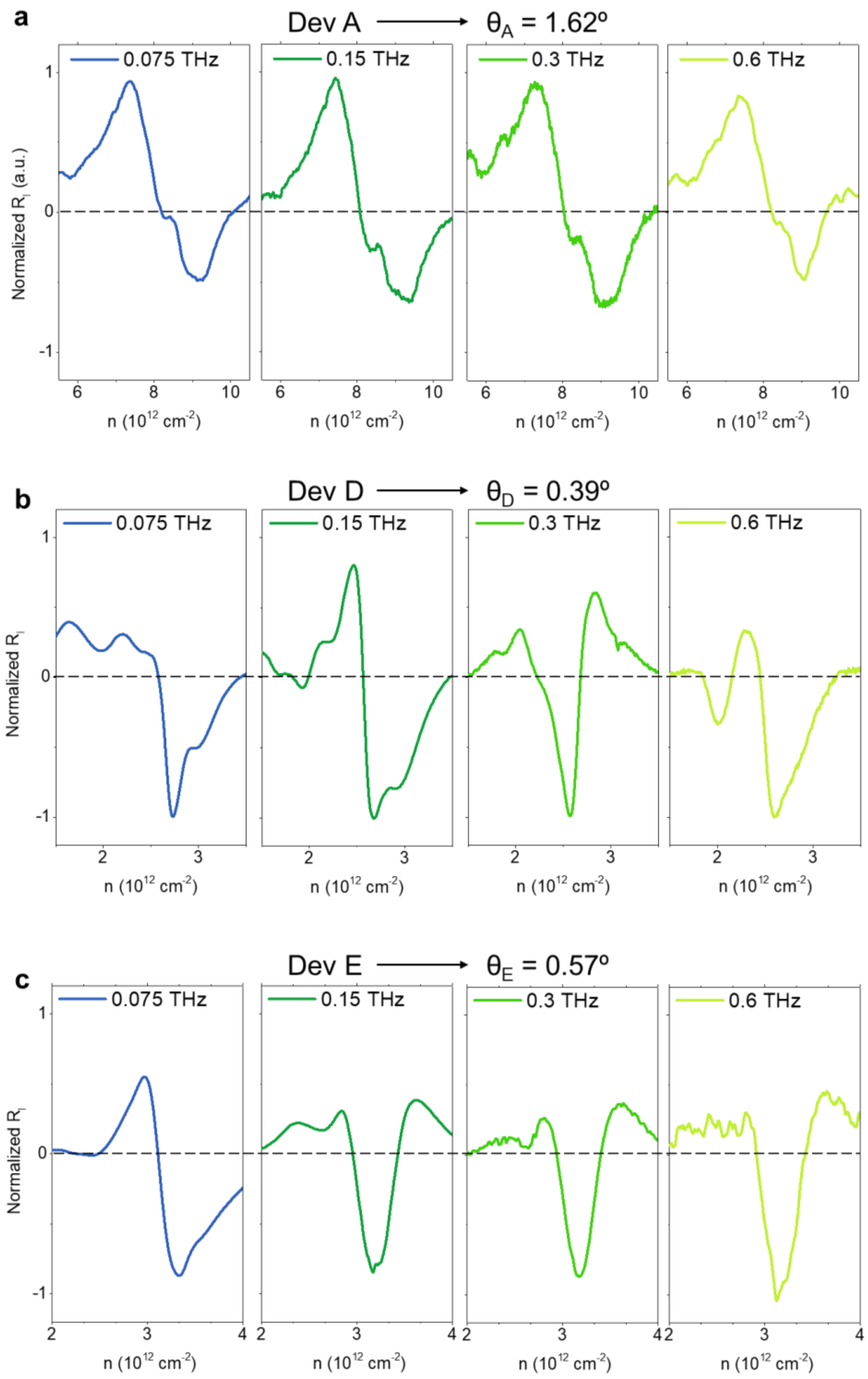

Figure S10. Low temperature photocurrent spectroscopy at the electron-band SDP. Normalized photocurrent responsivity R_I at sub-THz frequencies as a function of the carrier density, n, close to the electron-band SDP for **a**, device A, **b**, device D and **c**, device E. All measurements are undertaken at 10K

Note 9 – Additional calculations.

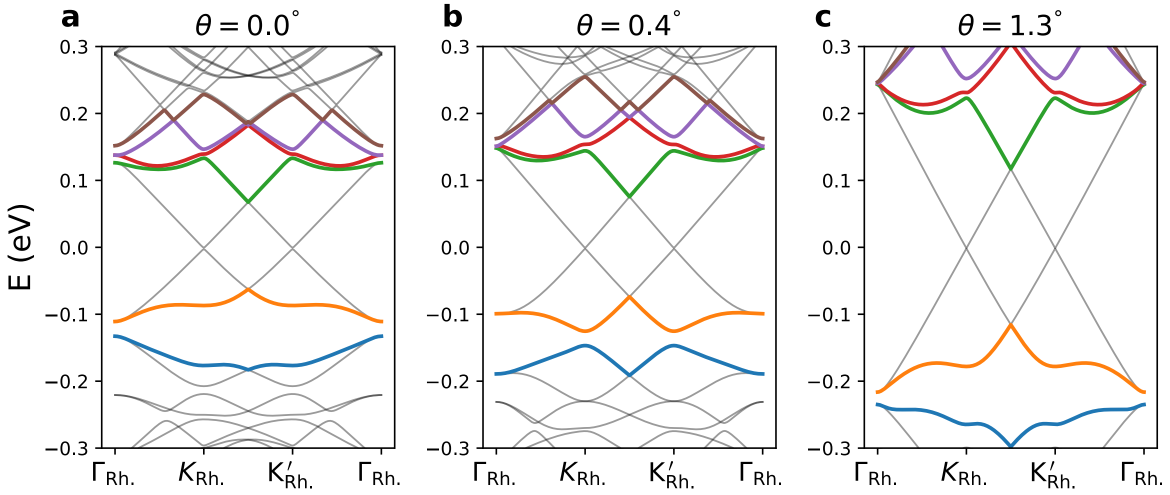

Figure S11. Band structures of graphene/hBN heterostructures with different twist angles. Electronic bands in a wider energy range around half-filling, showing the principal and secondary Dirac points in each case. The thicker, colored bands correspond to those relevant to transitions near the sDPs which are discussed in the main text. The three cases shown are **a,** untwisted, **b,** the case of $\theta = 0.44^{\circ}$ also shown in **Figures 5a** and **5b** of the main text, and **c**, a larger twist angle of $\theta = 1.3^{\circ}$.

Figure S11 shows the electronic bands near half-filling of three graphene/hBN systems with different twist angles. Panel **a** shows the untwisted case, in perfect agreement with similar calculations from Ref. 23. Panel **b** reproduces the bands for the $\theta = 0.44°$ case discussed in the main text, but now over a wider range of energies than those shown in the sDP zooms of **Figure 5a** and **Figure 5b**. Panel **c** shows the bands for the largest twist angle ($\theta = 1.3°$) for which energy gaps were extracted for Fig 4c. We note that the sDPs shift to higher energies as the twist angle increases, as noted previously[23]. We also note that the folding of graphene's Brillouin Zone (BZ) into the smaller BZ of the rhombohedral (Rh.) supercell used for tight-binding calculations can lead to an apparent change in the shape of the bands near the sDPs. This is particularly evident in the shapes of the blue and orange bands (labelled α and β in the main text) near the hole sDP. Although the K and K' points of graphene are folded to K and K' points of the rhombohedral BZ, they can swap places depending on the exact unit cell size. This leads to, for example, the smaller gap at the valence sDP (Δ_h^1), moving between $\Gamma_{Rh.}$ (panels **a** and **c**) and $K_{Rh.}$ (panel b) in these band structures.

Finally, we observe that only minute band gaps are found at the main DP in our model. The larger gap observed here in experiments require additional terms in the Hamiltonian to capture effects such as interaction and relaxation[24]. As these terms play a less prominent role at the sDPs, which are the main focus of this work, we have not included them in our calculations.

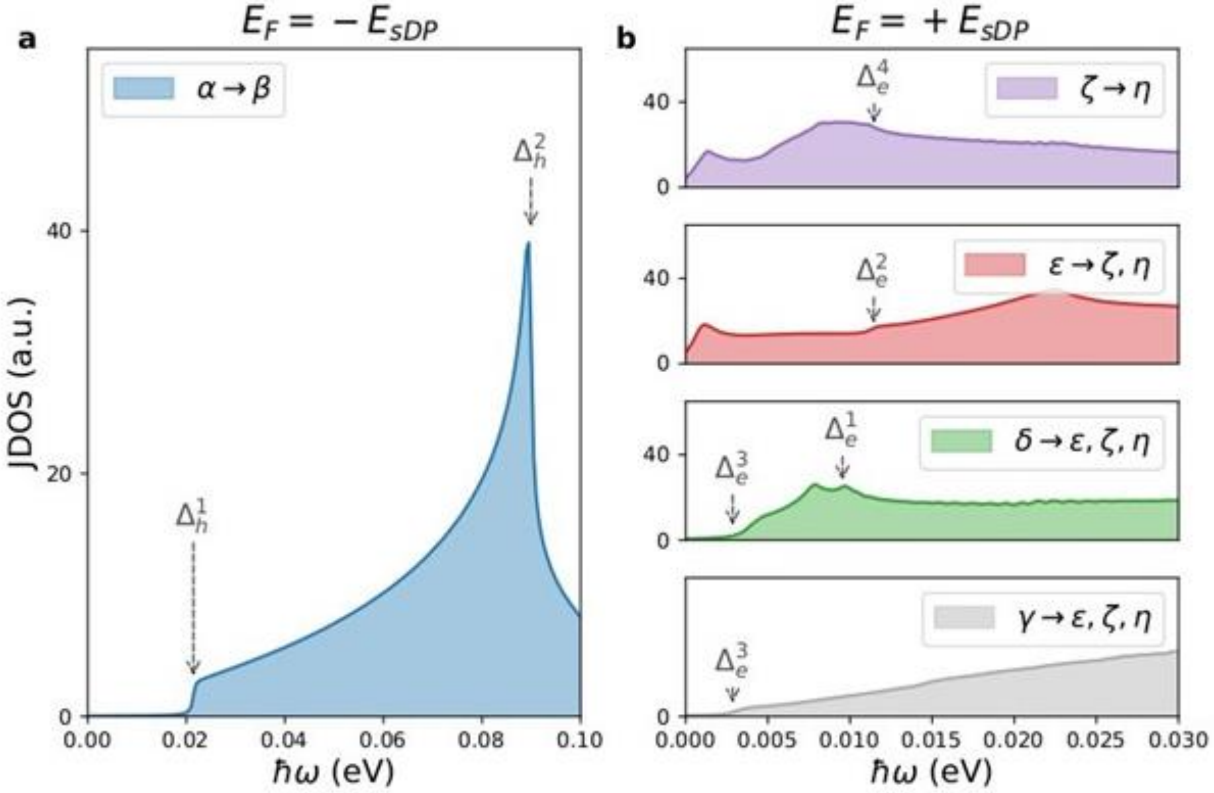

Figure S12. JDOS and interband conductivity of the system. a, JDOS between bands α and β (**Figure 5a,** main text), calculated as a function of the excitation energy $\hbar\omega$, corresponding to transitions near $E_F = -E_{sDP}$ (valence band sDP) for a device with θ=0.4˚. **b,** JDOS calculated between the different bands in **Figure 5b**, main text as a function of the excitation energy $\hbar\omega$, corresponding to transitions near $E_F = +E_{sDP}$. The legends show the bands included in the calculations.

Figure S12 shows the Joint Density of States (JDOS) corresponding to the possible band transitions and excitation energy ranges relevant to the **a**, valence and **b**, conduction sDP photoresponses discussed in **Figure 5** of the main text. The JDOS associated with transitions between a pair of bands *m* and *n*, with excitation energy ħω, is given by

$$\text{JDOS}_{(m\to n)}(\hbar\omega) = \int d\boldsymbol{k}\,\delta(\varepsilon_n(\boldsymbol{k}) - \varepsilon_m(\boldsymbol{k}) - \hbar\omega) \qquad \textbf{Eq. S10}$$

where $\varepsilon_n(\boldsymbol{k})$ is the energy of band *n* at a point ***k*** in reciprocal space. The legends in panels **a** and **b** indicate the bands considered in each panel, where we note that, due to the larger number of bands available, transitions to multiple bands are grouped together in panel **b**.

For Fermi energies near the valence sDP, the only relevant transitions are between the band directly below (α) and above (β) the gap. The onset of a finite JDOS in **Figure S12a** coincides with the minimum gap value between these bands (Δ_h^1), with a large peak observed at $\hbar\omega = \Delta_h^1$: this can

be associated with transitions near $\Gamma_{Rh.}$ where both bands are reasonably flat. Both the onset and peak in JDOS can also be clearly observed in the optical conductivity in panel **c**.

The situation is more complicated near the conduction side sDP, due to the larger number of bands which can play a role in transitions, including some (e.g. ε, ζ) which can play the role of either the valence (source) or conduction (destination) band, depending on the exact Fermi energy and $\boldsymbol{k}$ point. For clarity, in **Figure S12b** we group (and color) transitions in subpanels by their valence band. While there are features at excitation energies corresponding to the important energy gaps ($\Delta_e^1, \Delta_e^2, \Delta_e^3, \Delta_e^4$), they are less prominent than those near the valence side sDP, and are more difficult to directly connect to the optical conductivity (blue curve in panel **d.**). This can be understood by considering the following points:

- The optical conductivity is composed of all possible transitions at a particular excitation energy. The possible transitions overlap considerably in their energy ranges of possible excitation energies, so are harder to disentangle.
- Unlike the optical conductivity, which is for a fixed Fermi energy, JDOS captures all possible transitions between two bands. This is why the $\zeta \rightarrow \eta$ JDOS in the top panel of **Figure S12b** has finite contributions for $\hbar\omega < \Delta_e^4$, which corresponds to the gap between them at the conduction sDP. These two bands approach each other and eventually touch at higher energies, giving finite JDOS for smaller excitation energies which are irrelevant at $E_F \approx +E_{sDP}$.

The contribution of individual pairs of bands to the shift conductivity is discussed in the main text.

Note 10– Noise equivalent power.

We have evaluated the Noise-Equivalent-Power (NEP) in three of our detectors with θ <1° under excitation of THz at 10K. Since we have performed zero-bias photocurrent experiments, the Johnson–Nyquist noise, known as thermal noise, is the principal source of noise that we considered for the calculations. Thus, using Johnson–Nyquist relation for the noise spectral density, $N = \sqrt{4k_BTr_{ch}}$, the NEP can be calculated using the formula[8,10]:

$$NEP = \frac{\sqrt{4k_BTr_{ch}}}{R_I r_{ch}} \qquad \textbf{Eq. S11}$$

Interestingly, we observe an enhancement of the performance (i.e. lower values of NEP) in the vicinity of the SDPs in the examined moiré superlattices detectors with θ <1° (see **Figure S13**). We have quantified such reduction of the NEP by taking into account the minimum observable value of the NEP close to the main Dirac point ($NEP_{min,DP}$) and the minimum value of the NEP close to the satellite Dirac points ($NEP_{min,sDP}$). We have estimated an enhancement of the performance with ratios, $\delta NEP = NEP_{min,DP}/NEP_{min,sDP}$, down to ~0.2. This reduction of the

NEP demonstrates that graphene moiré superlattices devices can be used as sensitive and low noise THz detectors.

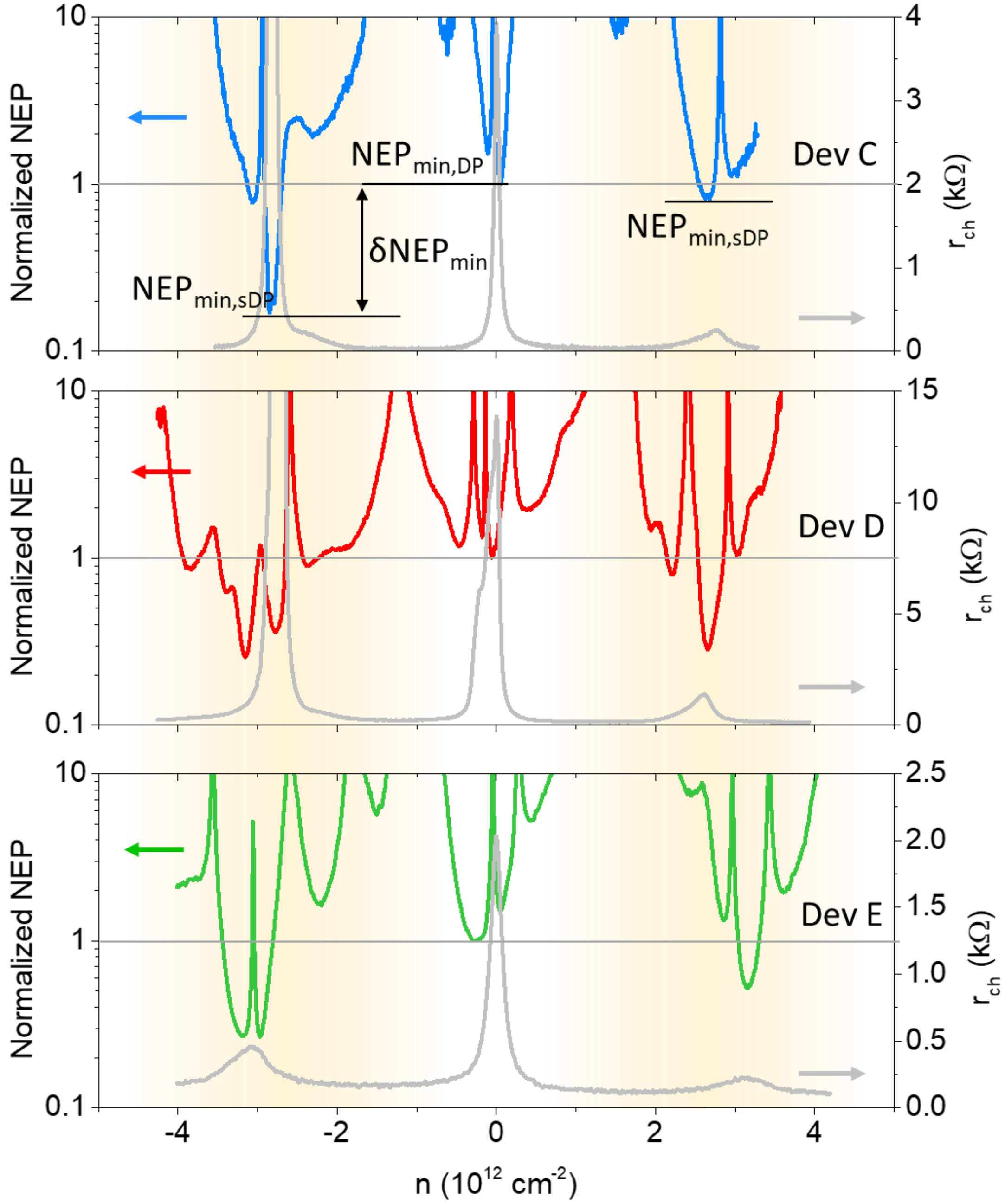

Figure S13. Low temperature Noise-equivalent-power of the moiré superlattices. Calculated NEP (Left) as a function of the carrier density, n, for the Devices C (top), D (middle) and E (bottom) at 10K under the excitation of 0.3 THz. NEP values have been normalized w.r.t. the minimum measured NEP close to the main Dirac point (horizontal grey line) for clarity. The corresponding device resistance curve (right) has been superimposed in the panels in grey color.

REFERENCES

1. Purdie, D. G. *et al*. Cleaning interfaces in layered materials heterostructures. *Nat Commun* **9**, 5387 (2018). https://doi.org/10.1038/s41467-018-07558-3

2. Gammelgaard, L. *et al*. Graphene transport properties upon exposure to PMMA processing and heat treatments. *2D Mater* **1** 035005 (2014). https://doi.org/10.1088/2053-1583/1/3/035005

3. Eckmann, A. *et al.* Raman Fingerprint of Aligned Graphene/h-BN Superlattices. *NanoLett* **13**, 5242–5246 (2013). https://doi.org/10.1021/nl402679b

4. A. Schäpers, A. et al. Raman imaging of twist angle variations in twisted bilayer graphene at intermediate angles. *2D Mater*. **9** 045009 (2022). https://doi.org/10.1088/2053-1583/ac7e59

5. Ribeiro-Palau, R. *et al*. Twistable electronics with dynamically rotatable heterostructures. *Science* **361**, 690–693 (2018). https://doi.org/10.1126/science.aat6981

6. Wallbank, J. R. et al. Excess resistivity in graphene superlattices caused by umklapp electron-electron scattering Nat Phys **15**, 32–36 (2019). https://doi.org/10.1038/s41567-018-0278-6

7. Caridad, J.M. et al. Room-Temperature Plasmon-Assisted Resonant THz Detection in Single-Layer Graphene Transistors. NanoLett. 24, 935−942 (2024) https://doi.org/10.1021/acs.nanolett.3c04300

8. Zak, A. *et al.* Antenna-Integrated 0.6 THz FET Direct Detectors Based on CVD Graphene. *Nano Lett* **14**, 5834–5838 (2014). https://doi.org/10.1021/nl5027309

9. Bandurin, D. A. *et al.* Dual origin of room temperature sub-terahertz photoresponse in graphene field effect transistors. *Appl Phys Lett* **112**, 141101 (2018). https://doi.org/10.1063/1.5018151

10. Rehman, A. *et al.* Temperature dependence of current response to sub-terahertz radiation of AlGaN/GaN and graphene transistors. *Appl Phys Lett* **121**, 213503 (2022). https://doi.org/10.1063/5.0129507

11. Tomadin, A., Tredicucci, A., Pellegrini, V., Vitiello, M. S. & Polini, M. Photocurrent-based detection of terahertz radiation in graphene. *Appl Phys Lett* **103**, 211120 (2013). https://doi.org/10.1063/1.4831682

12. Caridad, J.M. *et al*. Gate electrostatics and quantum capacitance in ballistic graphene devices. *Phys.Rev.B* **99**, 195408 (2019). https://doi.org/10.1103/PhysRevB.99.195408

13. Das Sarma, S., Adam, S., Hwang, E. H. & Rossi, E. Electronic transport in two-dimensional graphene. *Rev Mod Phys* **83**, 407–470 (2011). https://doi.org/10.1103/RevModPhys.83.407

14. Morozov, S. V *et al.* Giant Intrinsic Carrier Mobilities in Graphene and Its Bilayer. *Phys Rev Lett* **100**, 16602 (2008). https://doi.org/10.1103/PhysRevLett.100.016602

15. Dean, C. R. *et al.* Boron nitride substrates for high-quality graphene electronics. *Nat Nanotechnol* **5**, 722–726 (2010). https://doi.org/10.1038/nnano.2010.172

16. Hwang, E. H. & Das Sarma, S. Acoustic phonon scattering limited carrier mobility in two-dimensional extrinsic graphene. *Phys Rev B* **77**, 115449 (2008). https://doi.org/10.1103/PhysRevB.77.115449

17. Sunku, S. S. *et al.* Nano-photocurrent Mapping of Local Electronic Structure in Twisted Bilayer Graphene. *Nano Lett* **20**, 2958–2964 (2020). https://doi.org/10.1021/acs.nanolett.9b04637

18. Yankowitz, M. *et al.* Emergence of superlattice Dirac points in graphene on hexagonal boron nitride. *Nat Phys* **8**, 382–386 (2012). https://doi.org/10.1038/nphys2272

19. Yu, G. L. *et al.* Hierarchy of Hofstadter states and replica quantum Hall ferromagnetism in graphene superlattices. *Nat Phys* **10**, 525–529 (2014). https://doi.org/10.1038/nphys2979

20. Jung, M. & Shvets, G. Emergence of tunable intersubband-plasmon-polaritons in graphene superlattices. *Advanced Photonics* **5**, 026004 (2023). https://doi.org/10.1117/1.AP.5.2.026004

21. Ashcroft, N. W. & Mermin, N. D. Solid state physics. Holt-Saunders, (1976)

22. Sun, X. *et al*. Correlated states in doubly-aligned hBN/graphene/hBN heterostructures. *Nature Comm*. **12**, 7196 (2021). https://doi.org/10.1038/s41467-021-27514-y

23. Moon, P. & Koshino, M. Electronic properties of graphene/hexagonal-boron-nitride moiré superlattice. *Phys. Rev. B* **90**, 155406 (2014). https://doi.org/10.1103/PhysRevB.90.155406

24. Jung, J. et al. Moiré band model and band gaps of graphene on hexagonal boron nitride. Phys. Rev. B 96, 85442 (2017). https://doi.org/10.1103/PhysRevB.96.085442
